# Pseudonajide peptide derived from snake venom alters cell envelope integrity interfering on biofilm formation in *Staphylococcus epidermidis*

**DOI:** 10.1186/s12866-020-01921-5

**Published:** 2020-08-03

**Authors:** Rafael Schneider, Muriel Primon-Barros, Rafael Gomes Von Borowski, Sophie Chat, Sylvie Nonin-Lecomte, Reynald Gillet, Alexandre José Macedo

**Affiliations:** 1 Université de Rennes, CNRS, Institut de Génétique et Développement de Rennes (IGDR), UMR 6290 Rennes, France; 2 Laboratório de Biofilmes e Diversidade Microbiana, Faculdade de Farmácia and Centro de Biotecnologia, Universidade Federal do Rio Grande do Sul, Porto Alegre, Brazil; 3 Faculté de Pharmacie, Université de Paris, CNRS, CiTCoM, UMR 8038 Paris, France

**Keywords:** Antimicrobial peptide, *Staphylococcus epidermidis*, Biofilm, Snake venom, Pseudonajide

## Abstract

**Background:**

The increase in bacterial resistance phenotype cases is a global health problem. New strategies must be explored by the scientific community in order to create new treatment alternatives. Animal venoms are a good source for antimicrobial peptides (AMPs), which are excellent candidates for new antimicrobial drug development. Cathelicidin-related antimicrobial peptides (CRAMPs) from snake venoms have been studied as a model for the design of new antimicrobial pharmaceuticals against bacterial infections.

**Results:**

In this study we present an 11 amino acid-long peptide, named pseudonajide, which is derived from a *Pseudonaja textilis* venom peptide and has antimicrobial and antibiofilm activity against *Staphylococcus epidermidis*. Pseudonajide was selected based on the sequence alignments of various snake venom peptides that displayed activity against bacteria. Antibiofilm activity assays with pseudonajide concentrations ranging from 3.12 to 100 μM showed that the lowest concentration to inhibit biofilm formation was 25 μM. Microscopy analysis demonstrated that pseudonajide interacts with the bacterial cell envelope, disrupting the cell walls and membranes, leading to morphological defects in prokaryotes.

**Conclusions:**

Our results suggest that pseudonajide’s positives charges interact with negatively charged cell wall components of *S. epidermidis,* leading to cell damage and inhibiting biofilm formation.

## Background

Several animals in different phyla in the animal kingdom have developed the ability to produce venoms, including annelids (e.g. bearded fireworm), arthropods (e.g. bees, scorpions, and spiders), cnidarians (e.g. sea anemones), echinoderms (e.g. sea urchins), mollusks (e.g. cone snails) and vertebrates (e.g. snakes and mammals) [1, 2]. Animal venoms are complex mixtures of inorganic salts, small organic molecules, high-molecular-weight proteins including enzymes, and peptides, used for both protection and predation [2, 3]. Because they are both potent and specific in their interactions with the cell wall and membrane components of different cells, venom constituents are attractive candidates for the development of novel therapeutics and pesticides [4], although they remain largely unexplored.

The rich blend of molecules found in venoms contains many antimicrobial peptides (AMPs) [1], and AMP sequences are available in various databases (such as http://aps.unmc.edu/AP) [3]. AMPs have complex and wide-ranging mechanisms of action. They can directly target bacterial membranes, damaging cell integrity and consequently causing osmotic imbalance. They can also disrupt macromolecular synthesis, interfering in cell wall biosynthesis [5]. Due to this complexity and the widescale of their target interactions, resistance to such molecules seems to arise less commonly than with conventional antibiotics [6].

Cathelicidin-related antimicrobial peptides (CRAMPs) from snake venoms (specially from Elapidae and Viperidae families) have been studied as models for the design of new antimicrobial pharmaceuticals against bacterial infections, facilitated by complete genome sequences available [7]. These bioactive molecules are structurally characterized by an N-terminal segment with a gene-encoded signal peptide and a cathelin domain derived from the cathepsin L-inhibitor (pro-peptide), followed by a C-terminal antimicrobial domain with diverse structures (mature peptide) [8]. Venom cDNA libraries from *Naja atra, Bothrops atrox, Crotalus durissus terrificus, Pseudonaja textilis, Ophiophagus hannah,* and *Bungarus fasciatus* snake species have revealed different types of cathelicidins (NA-CATH, batroxicidin, crotalicidin, Pt_CRAMP1, OH-CATH, OH-CATH30, cathelicicin-BF) with remarkable antimicrobial activity against Gram-positive and -negative bacteria, as well as fungi [1, 9, 10].

About 75% of human microbial infections are promoted by biofilm formation, which is increasingly recognized by the public health community as an important source of pathogens, especially in medical device-associated and persistent infections [11]. Biofilms are well-organized microbial associations normally attached to abiotic or biotic surfaces. Their structure is characterized by matrix accumulation, and the formation process consists of several stages (cell adherence, microcolonies formation, biofilm maturation and dispersion) [12, 13]. There are several advantages to this form [14]: a biofilm community is difficult to treat due to the physical barrier against antibiotics and immune system factors [15]; and the structure is related to much higher antimicrobial resistance [16]. In most cases, the first treatment option for biofilm on medical devices is removal of the devices themselves, leading to increases in both patient suffering and health system financial expenditures [17].

*Staphylococcus epidermidis* is a commensal microorganism widely present on human skin [18], making this species one of the main causes of infections related to biofilm formation in medical devices [19, 20]. Indeed, biofilm formation is the most prominent virulence factor during the pathogenesis of *S. epidermidis* [21]. Commensal skin flora or hospital bacteria can adhere to a foreign body, replicate, and form a biofilm, which can then for instance invade peri-implant tissue, causing serious infection [17, 22]. *S. epidermidis* infections on central venous catheters occur annually in approximately 80,000 cases in the United States, leading to several blood infection cases [23, 24]. Furthermore, *S. epidermidis* is related to 17–39% of infections in prosthetic valve endocarditis [25]. Due to this prevalence, and the global increase in bacterial resistance, the search for new molecules to treat bacterial infection is extremely urgent [26].

Here, we present pseudonajide, a synthetic peptide made up of 11 amino acids and derived from *Pseudonaja textilis* snake venom. The peptide possesses antimicrobial and antibiofilm activity against *S. epidermidis*, and our results suggest that it acts on this bacteria’s cell wall and membrane components quite quickly and at low doses.

## Results

### Snake venom antimicrobial peptides present highly conserved motifs

**Fig. 1:**
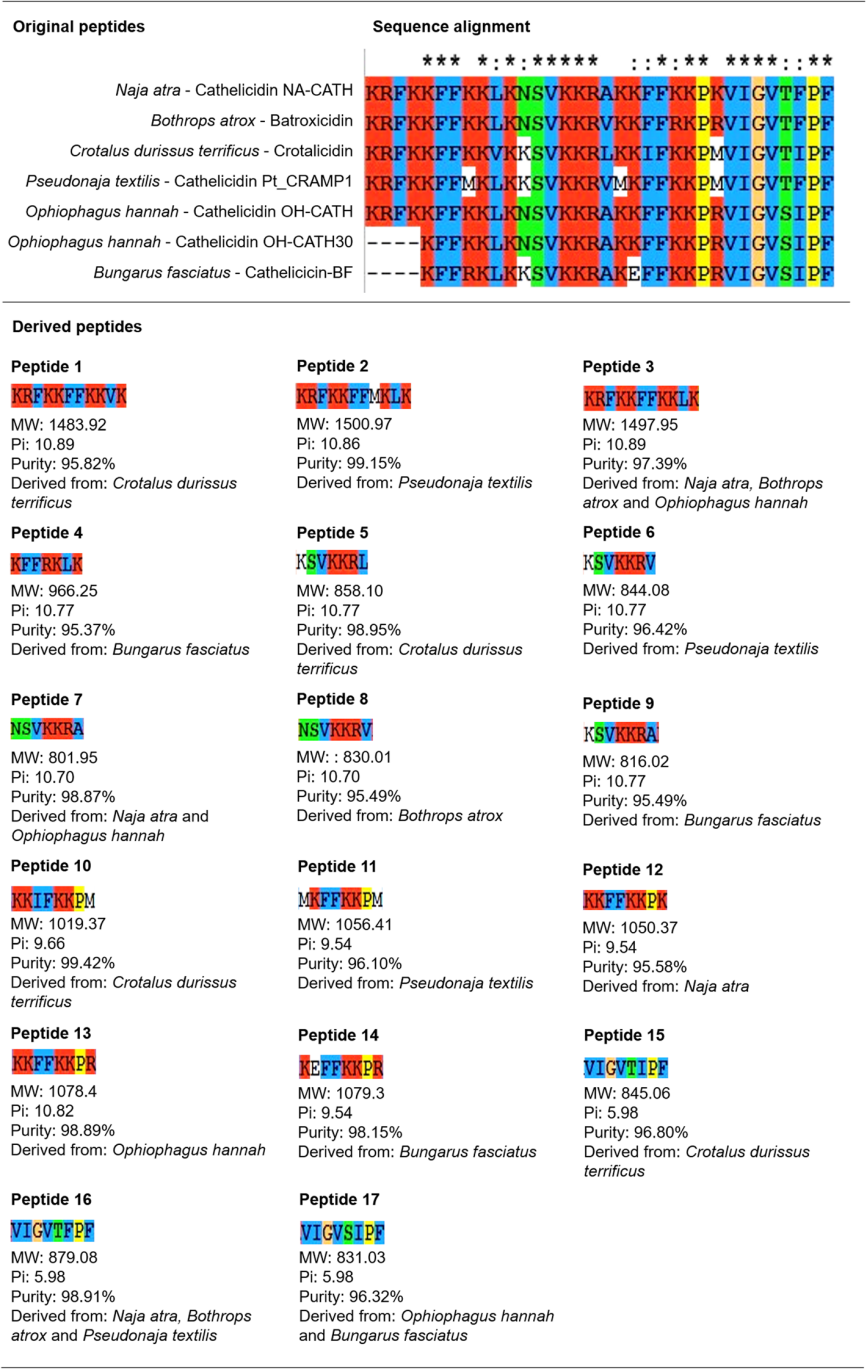
Peptide alignment and selection of snake venom derived peptides. (Top) Chart demonstrating the sequence alignment of seven peptides derived from snake venom. (Bottom) After analysis with Clustal X software [27], 17 sequences of 7, 8 or 11 amino acids and different charges and hydrophobicity were selected for antibiofilm and antimicrobial testing. The figure shows each snake venom derived peptide with respective molecular weight (MW), isoelectric point (Pi), purity, and originating snake species

A total of 170 antimicrobial peptide sequences from ant, bee, centipede, cone snails, scorpion, snake, spider or wasp venoms were analyzed. Sequence alignments within each taxonomic group were performed using ClustalX (Fig. 1 and Additional files 1, 2, 3, 4, 5, 6 and 7). The alignment of snake sequences (*Naja atra* - Cathelicidin NA-CATH; *Bothrops atrox* - Batroxicidin; *Crotalus durissus terrificus* - Crotalicidin; *Pseudonaja textilis* - Cathelicidin Pt_CRAMP1; *Ophiophagus hannah* - Cathelicidin OH-CATH; *Ophiophagus hannah* - Cathelicidin OH-CATH30, and; *Bungarus fasciatus* - Cathelicicin-BF) revealed a greater number of conserved motifs among snake peptides than that found in other groups. Subsequently, 17 conserved amino acid sequences and their close variants (Fig. 1) were selected as derived peptides for antibiofilm and antimicrobial activity evaluations.

### Peptides 1, 2, and 3 have antibiofilm activity in *S. epidermidis*

**Fig. 2:**
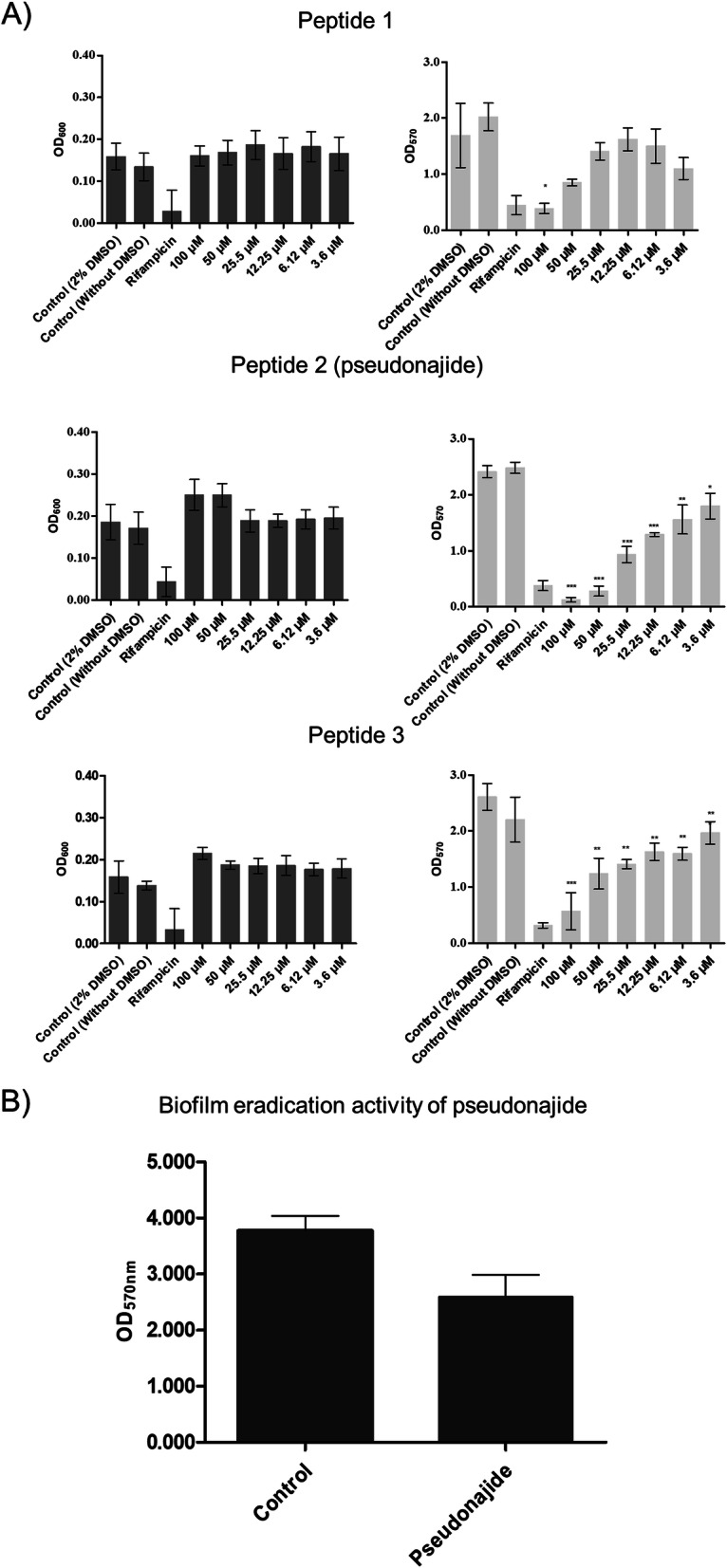
Antibiofilm and biofilm eradication activity. (A) Antibiofilm formation (left graphs) and bacterial growth (right graphs) after 24 h of exposure to different concentrations of peptide 1, pseudonajide (peptide 2), or peptide 3. *S. epidermidis* ATCC 35984 cell suspension was incubated alone or in the presence of decreasing concentrations of peptides. An adapted crystal violet protocol was used for biomass measurement at optical density of 570 nm (OD_570_) [29], and growth analysis was done at an optical density of 600 nm (OD_600_). Culture medium with 2% dimethyl sulfoxide (DMSO), and culture medium alone were used as growth control, while rifampicin was the antibiofilm positive control. OD_600_ was measured at time zero and at 24 h for growth normalization. (B) The biofilm eradication activity of pseudonajide (peptide 2), pseudonajide (bottom graph) was measured by adding 25 μM of pseudonajide in pre-formed biofilm and incubating for further 24 h. All the tests were performed with at least 3 different biological replicates each including at least 3 technical replicates. Error bars are shown, and statistical analysis was performed using Student’s *t*-test, where: *, *p* < 0.05; **, *p* < 0.01; ***, *p* < 0.001

The first aim of this work was to perform a screening for antibiofilm activity in 17 synthetic small peptides derived from snake venom sequences. For that, we chose two different species of bacteria, one Gram-negative (*Pseudomonas aeruginosa* PAO1), and one Gram-positive (*S. epidermidis* ATCCC 35984). The selection took into account their biofilm production capabilities, and their recognized value as good models for the study of biofilm formation and structure [12, 28]. For screening, a crystal violet stain protocol was used in the absence or presence of peptides at different concentrations. No effects were detected on biofilm formation by *P. aeruginosa* (Additional file 8). On the other hand, peptides 1, 2, and 3 demonstrated strong activity against *S. epidermidis* biofilm. After 24 h of exposure to different concentrations, there was a considerable reduction in biofilm mass (Fig. 2a). At a concentration of 100 μM, the biofilm mass was reduced by 77, 95, and 78% for peptides 1, 2, and 3, respectively (Fig. 2a).

Peptide 2 demonstrated greater antibiofilm activity than peptides 1 and 3. The considerable reduction of 63% in biofilm formation in the presence of 25 μM of peptide 2 led us to select that particular molecule at that specific concentration for the following experiments. We named the peptide “pseudonajide” after the name of the snake it was derived from, *Pseudonaja textilis*. In order to test its biofilm eradication activity, we pre-cultured *S. epidermidis* cells for 24 h, adding pseudonajide to pre-formed biofilm and incubating for another 24 h. The final quantification of biofilm mass showed a reduction of about 30% in the presence of the molecule (Fig. 2b).

### Pseudonajide has antimicrobial activity against *S. epidermidis*

**Fig. 3:**
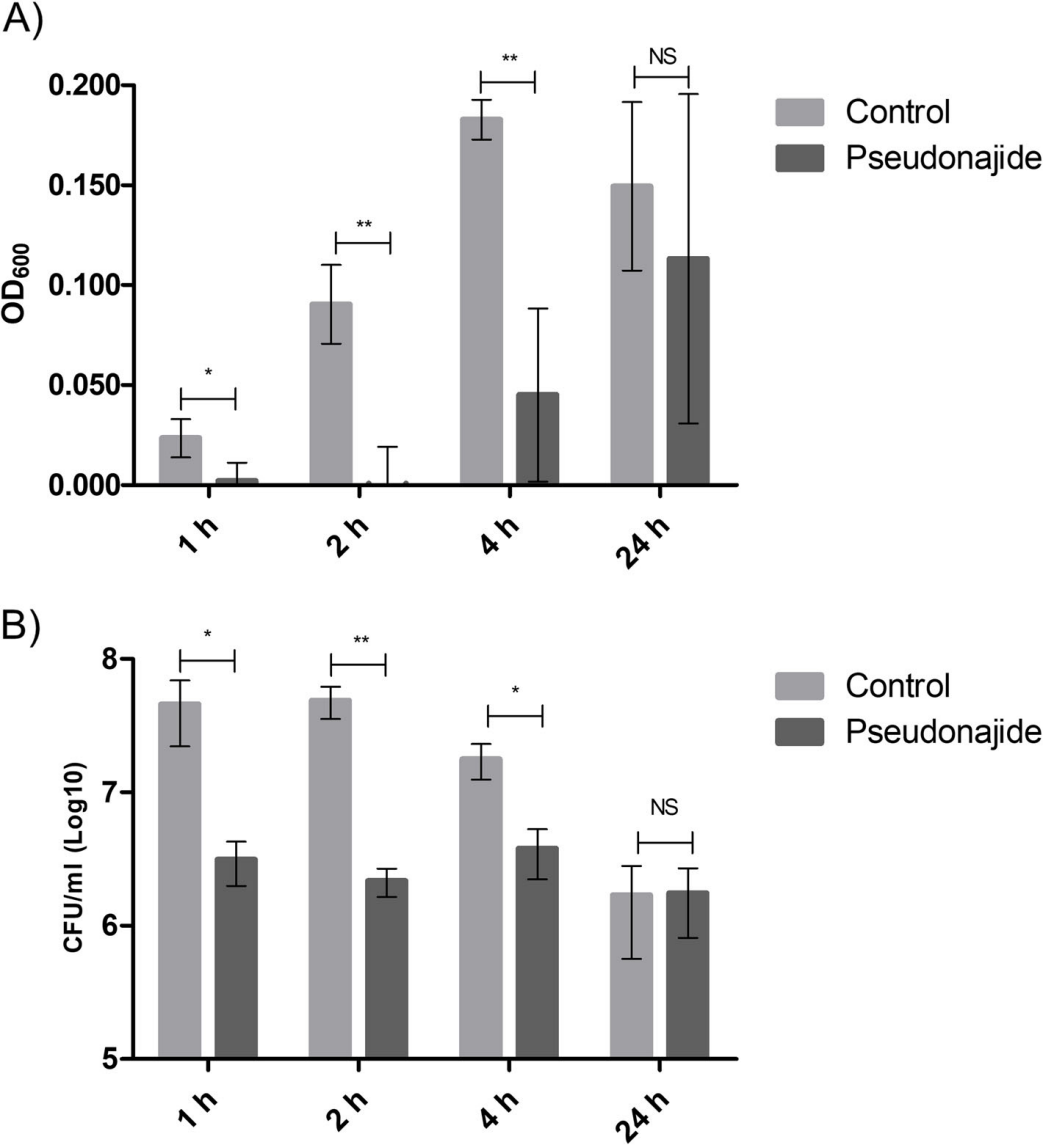
Antimicrobial activity of pseudonajide (peptide 2). *Staphylococcus epidermidis* was cultured in the same conditions as the biofilm tests, but with a different pseudonajide concentration (25 μM). **a** Optical density measurements at 600 nm (OD_600_) show a reduction in bacterial growth in the presence of 25 μM pseudonajide (peptide 2) after 1, 2, and 4 h. **b** Colony-forming unit (CFU) testing confirmed the reduction in cell viability as compared to the control. All the tests were performed with at least 3 different biological replicates, each having at least 3 technical replicates. Error bars are shown, and statistical analysis was performed applying Student’s *t*-test, where: *, p < 0.05; **, *p* < 0.005; NS, non-significant

We decided to test the antimicrobial activity over a shorter period of time, because no difference had been observed after 24 h. Growth and colony-forming unit (CFU) tests were performed. Cells were incubated in the same conditions as for the antibiofilm tests, with or without 25 μM pseudonajide. After 1, 2, 4, and 24 h incubation, we measured the optical density at 600 nm (OD_600_) and assessed the CFU counts. Figure 3 shows clearly that the molecule caused a huge decrease in bacterial growth as compared to the control. Accordingly, the number of viable cells determined by CFU counts decreased after 1, 2, or 4 h of incubation with pseudonajide.

### Pseudonajide binds to the cell wall and membrane, causing permeabilization

**Fig. 4:**
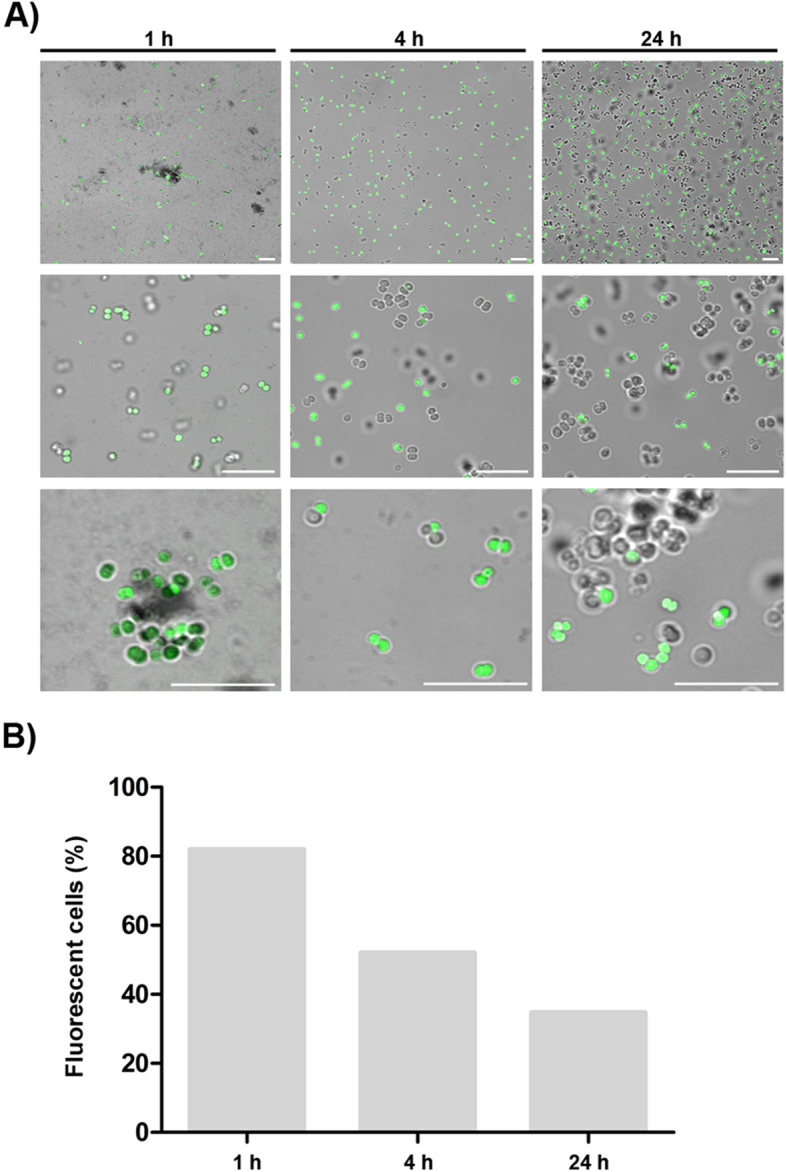
Pseudonajide is located on the cell envelope and inside the bacterial cell. **a** Confocal microscopy images of *Staphylococcus epidermidis* incubated for 1, 4, and 24 h with 25 μM pseudonajide tagged with fluorescein isothiocyanate (FITC). Scale, 10 μm. Cells were washed once with saline solution, and 3 μL cell suspension were added to each glass slide. **b** Graph demonstrating the percentage of cells that were fluorescent at each time point. Shown are 5 random fields, with approximately 1000 cells counted for each time point. Calculated by dividing the number of fluorescent cells by the total number of cells in the field. Images were analyzed using Fiji software

To better understand pseudonajide’s binding site, we synthesized peptides tagged with fluorescein isothiocyanate (FITC) for use in confocal microscopy experiments. Cells were incubated with 25 μM FITC-tagged pseudonajide for 1, 4, or 24 h. After incubation, confocal microscopy showed that the molecule was located around or inside the bacterial cell, but not in the biofilm matrix (Fig. 4). Another important finding was the reduction in the number of fluorescent cells over time, with decreased peptide-tagged cell counts after 4 h and 24 h incubation.

**Fig. 5:**
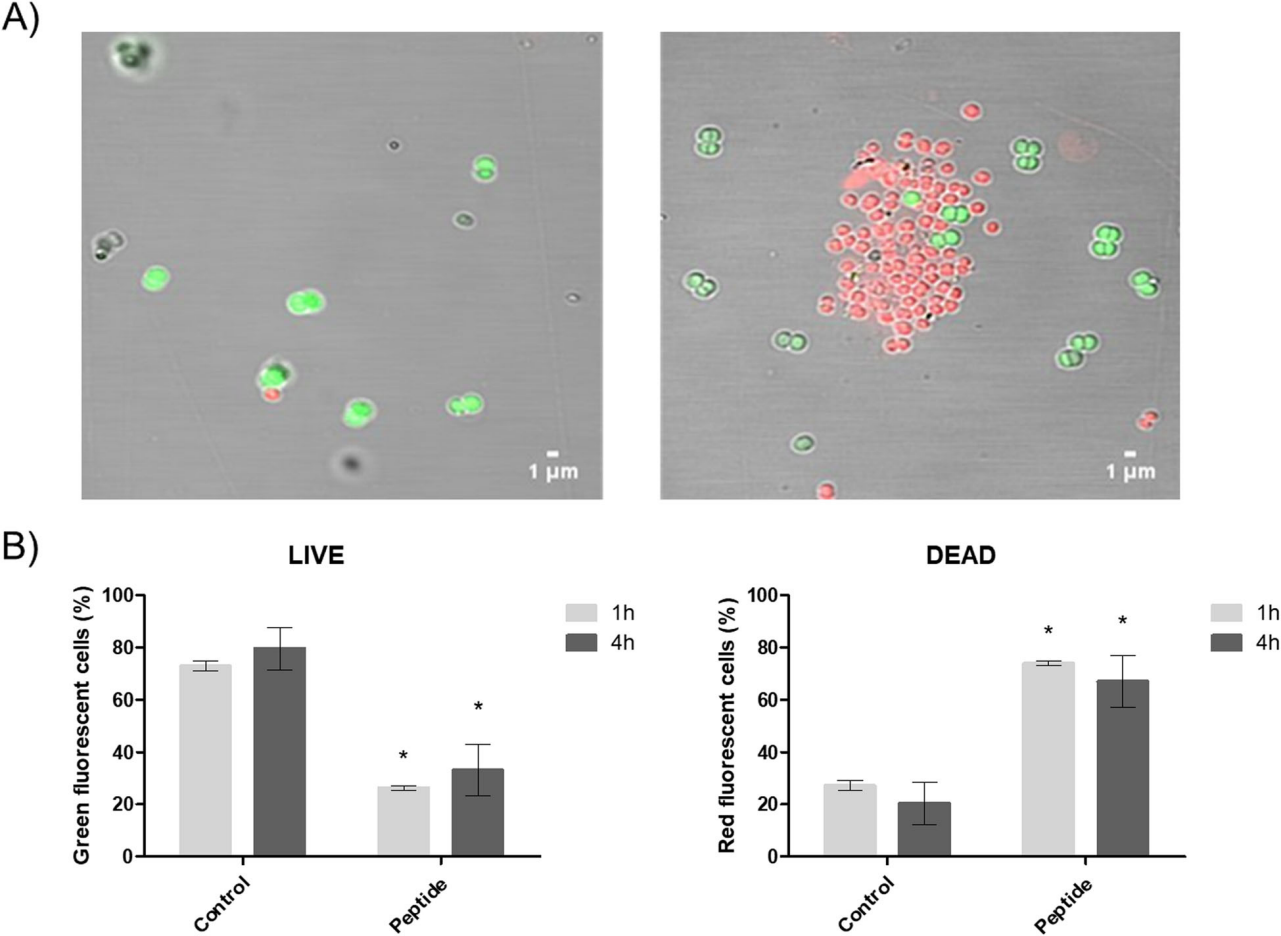
LIVE/DEAD experiments in the presence of pseudonajide. **a** Confocal microscopy images showing cells (left) unexposed and (right) exposed to the molecule. After incubation, cells were treated with LIVE/DEAD *Bac*Light reagent (Thermo Fisher). **b** Graph of the percentages of DEAD (red) and LIVE (green) cells. At least 1000 cells were counted in more than 10 randomly chosen fields per condition, and the experiments were performed in triplicate for each time point. Bars represent the standard deviation. Statistical analysis comparing results to the respective control conditions (Student’s *t-*test): *, *p* < 0.05

To confirm that the interaction occurs between pseudonajide and *S. epidermidis* cell wall and membrane, we did LIVE/DEAD experiments. It is demonstrated in the literature that propidium ions can enter cells with high membrane potential [30]. Cells were cultured for 4 h with or without 25 μM pseudonajide. Confocal microscopy image analysis demonstrated an increase in cell death when in the presence of pseudonajide. Moreover, statistical analysis shows that there was a significant decrease in the number of impermeable cells (green) when the peptide is present (Fig. 5). These data suggest that pseudonajide is interfering with cell wall and membrane integrity.

### Pseudonajide damages *S. epidermidis* cell wall and membrane

**Fig. 6:**
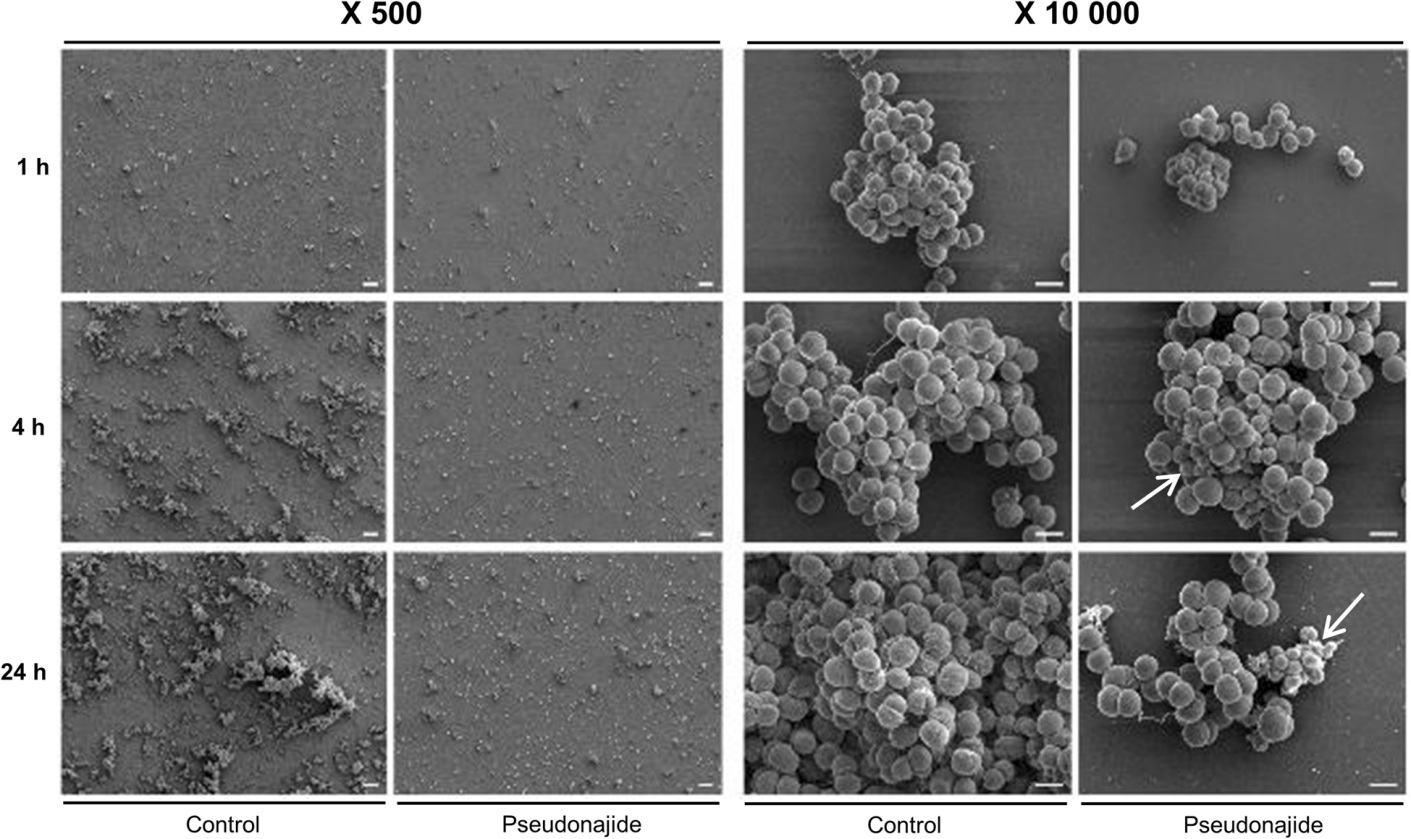
Scanning electron microscopy images of *Staphylococcus epidermidis* in the presence or absence of pseudonajide. Bacterial cells were cultured with or without 25 μM pseudonajide (peptide 2) for 1, 4, and 24 h. Plastic slides were placed inside the culture well plates and used to analyze biofilm and cell morphology. White arrows indicate the cells with different morphologies at 4 and 24 h in the presence of pseudonajide. Images are shown at 500 and 10,000x magnification, and were obtained using a JEOL JSM-7100F scanning electron microscope

**Fig. 7:**
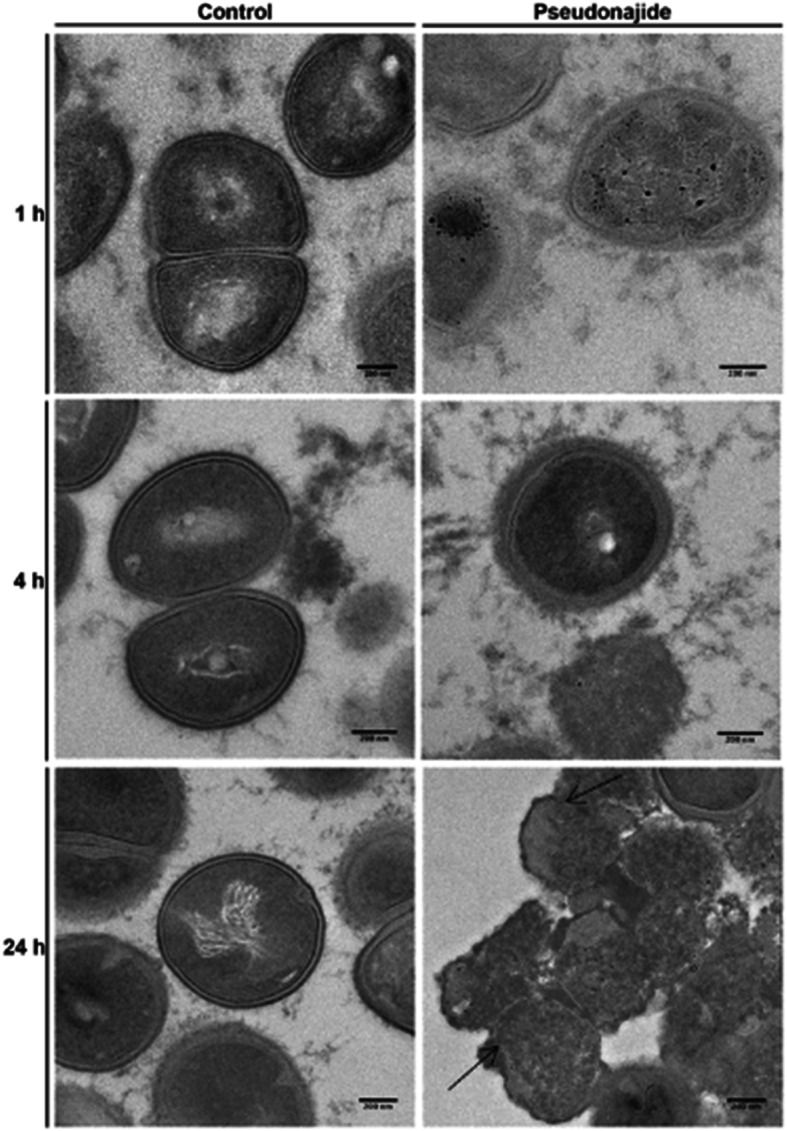
Transmission electron microscopy images of *Staphylococcus epidermidis* in the presence or absence of pseudonajide. *S. epidermidis* cells were cultured with or without 25 μM of the peptide and imaged at three different time points. Cells were detached from the well plate using disposable tips, and all content was treated following the TEM protocol (Materials and Methods). Dark arrows indicate cell wall defects and membrane disruption in the presence of pseudonajide. Scale bars, 200 nm

To check for morphological changes in *S. epidermidis* cells after exposure to the peptide, microscopy experiments were then performed after 1, 4, and 24 h incubation with or without 25 μM pseudonajide. We chose to approach this in two distinct ways, using both scanning electron microscopy (SEM) and transmission electron microscopy (TEM). The SEM experiments were performed by culturing the cells in the same conditions as before, with plastic slides added to the culture well for cell adherence. Our most notable result was that after 4 and 24 h incubation, cell adhesion was much weaker when cultured with the peptide, although no difference was observed after just 1 h (Fig. 6). Another important characteristic we noted was that several cells exposed to this molecule had a shrunken morphology and were smaller than non-exposed cells (Fig. 6, white arrows). Again, this morphology was only noted after 4 h or 24 h incubation. A final point that must be highlighted is that some extravasated material was present surrounding the shrunken cells, as was apparent in the SEM images (Fig. 6, white arrows). None of these phenomena were observed in untreated cultures. After the SEM experiments and analysis, two questions remained unanswered: how does pseudonajide causes cells to shrink? Is there any damage to the cell wall or to the membrane? To address these questions, we performed TEM. This imaging method allows for the analysis of cell component ultrastructures and thus the analysis of cell wall and membrane integrity. Analysis of the resulting images demonstrated disrupted cells after pseudonajide exposure (Fig. 7, dark arrows). Specifically, after 4 and 24 h of peptide exposure, the cell wall is not intact, and the cell sizes are completely different than those of the control. Moreover, cytoplasmic material looks condensed in peptide-exposed cells. (Fig. 7).

### Pseudonajide increases the expression of genes coding for teichoic acid synthesis

The results obtained from microscopy analysis led us to hypothesize that pseudonajide acts on cell walls and membranes. Indeed, cationic peptides are known to be able to interact with the cell wall of Gram-positive bacteria [31] and to influence membrane fluidity when engaging with the phospholipid bilayer [32]. One of the first molecules that is supposed to interact with cationic peptides is teichoic acid, a negatively charged molecule present in Gram-positive cell walls [33]. To investigate this, real-time quantitative PCR tests were done, with *S. epidermidis* cultured in the same conditions as the previous experiments.

**Fig. 8:**
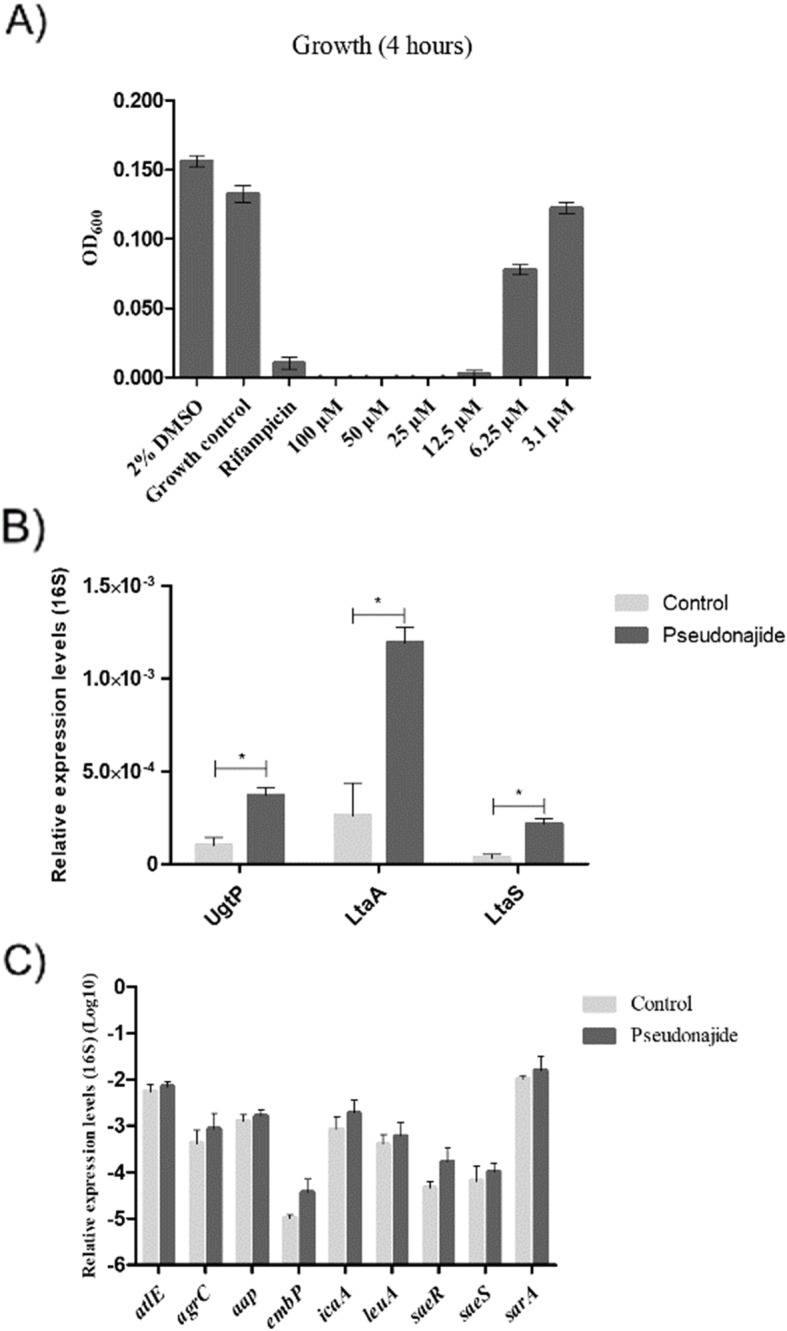
Gene expression analysis. Graphs of gene expression analysis for three genes coding for the lipoteichoic acid assembly cascade. **a** Bacterial growth after 4 h exposure to different concentrations of pseudonajide (peptide 2). Culture medium with 2% DMSO was used as a control, and rifampicin was the antibiofilm positive control. OD_600_ was measured at time zero and at 24 h for growth normalization. For gene expression analysis, cDNA was obtained through reverse transcriptase reactions on mRNA extracted from *Staphylococcus epidermidis* cultured in the presence (peptide) or absence (control) of 6.25 μM pseudonajide. **b** Expression levels of genes *ugtP, ltaA,* and *ltaS* under the same testing conditions. **c** Expression levels of biofilm-related genes under these same conditions. The 2^-ΔΔct^ method was used to normalize expression levels to 16S rRNA [34]. All tests were performed in at least 3 different biological replicates each having at least 3 technical replicates. Error bars are shown, and statistical analysis was performed using Student’s *t*-test, where: *, *p* < 0.05

Upon testing 4 h incubation with serially diluted doses of pseudonajide (3 μM to 100 μM), the concentration of 6.25 μM, sufficient to inhibit 50% of bacterial growth (Fig. 8), was selected for gene expression studies. Three genes involved in teichoic acid biosynthesis (UgtP, LtaA and LtaS) were tested and showed increased expression levels in the presence of sub-lethal concentration of pseudonajide (Fig. 8b). These results led us to hypothesize that pseudonajide interacts with teichoic acid in the *S. epidermidis* cell wall, causing a strong interaction with this structure, and leading to cell permeability. The same extracted RNA sample was used for expression analysis of nine biofilm-related genes: *atlE*, *agrC*, *aap*, *embP*, *icaA*, *leuA*, *saeR*, *saeS*, and *sarA*. No significant differences in expression were observed between control and peptide-treated conditions for these nine genes (Fig. 8c).

### Pseudonajide interacts with lipoteichoic acid (LTA) in vitro

**Fig. 9:**
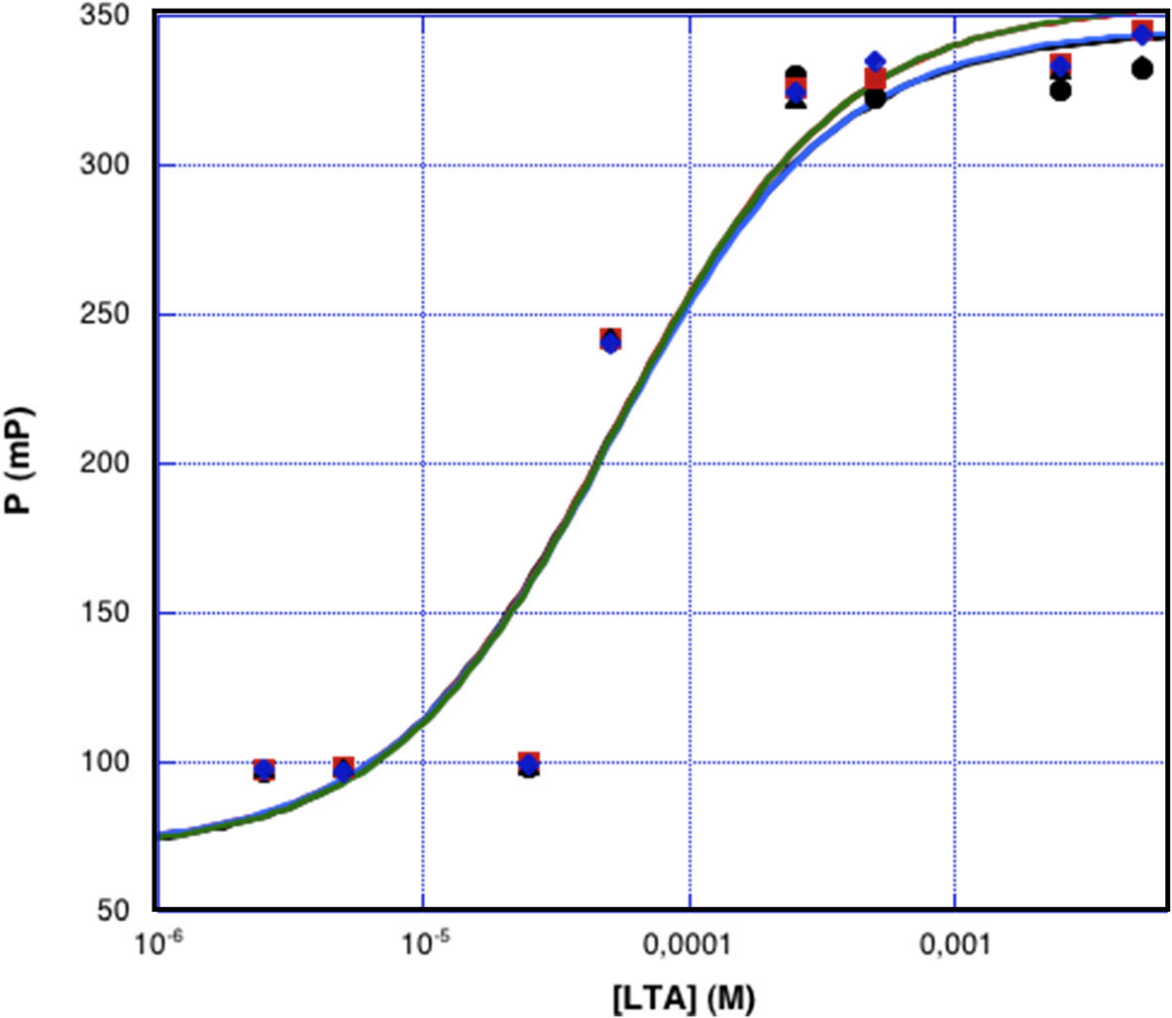
Interaction between pseudonajide and LTA. The Klotz plot of the fluorescence polarization vs LTA concentration indicates the interaction between FITC-pseudonajide and LTA. Measurements were recorded in water for a FITC-pseudonajide constant concentration of 5 μM and LTA monomer concentrations corresponding to 0.5, 1, 5, 10, 50, 100, 500 and 1000 M equivalents. Titration series were set in triplicates. Data points arrange along with a sigmoid. Fitting according to a 1:1 model discloses a mid-titration corresponding to a Kd in the range of 47-51 μM. The sharper and steeper rise of the sigmoid is evocative of a cooperative binding process

To probe the interaction between pseudonajide and LTA, we performed binding experiments by fluorescence polarization (FP) using FITC-tagged pseudonajide. Peptide concentration was set constant to 5 μM while LTA monomer concentration varied from 2.5 μM to 5 mM. Titration series was set in triplicates. FITC-pseudonajide FP increased with LTA concentration, an indication of binding between the two molecules (Fig. 9). The LTA concentration range used allowed us to observe an almost complete titration curve. Mid titration occured at around 50 μM LTA. The sigmoid rise is sharper than expected for a 1:1 binding model, indicating cooperative binding of more than one molecule of LTA per peptide. Albeit observed in vitro, these results strongly support the conclusion derived from microscopy and RT-qPCR that pseudonajide interacts with the cell walls and membranes of Gram-positive bacteria by binding to lipoteichoic and teichoic acids. This would also explain why the peptide is not active against Gram-negative bacteria like *Pseudomonas aeruginosa*.

### Pseudonajide is not cytotoxic to human cells

**Fig. 10:**
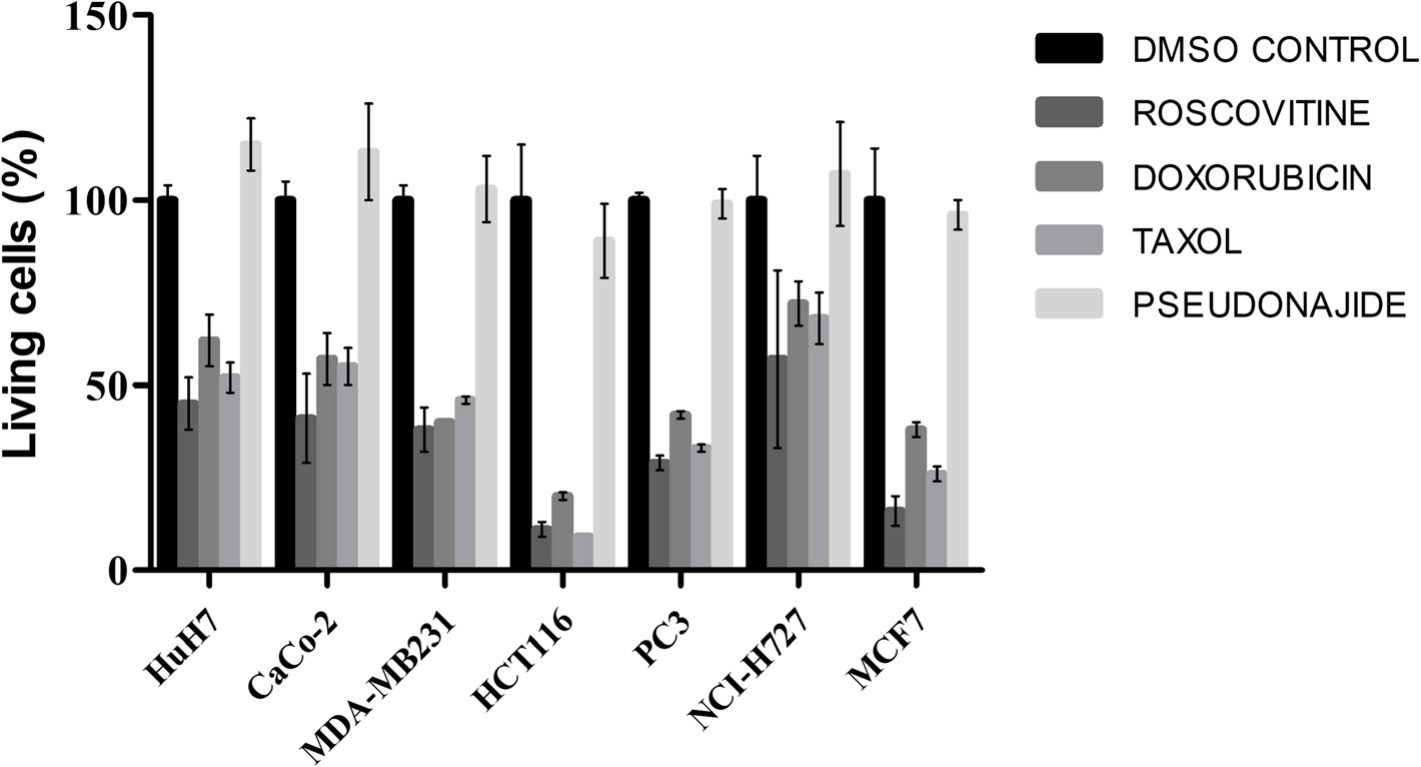
Evaluation of pseudonajide cytotoxicity. Graph demonstrating the percentage of living cells after 24 h exposure to 25 μM of each peptide. For the tests, seven different human cell lines were used. For comparison, the number of living cells in the DMSO control was considered as 100%, and the three cytotoxic drugs roscovitine, doxorubicin, and taxol were used as positive controls. All tests were performed at the ImPACcell automatized platform of cytotoxicity evaluation (Université de Rennes 1)

One of the main challenges in the development of antimicrobial peptides as therapeutics is their potential toxicity to human cells [35, 36]. We therefore performed toxicity tests using seven human cell lines: HuH7 (hepatocellular carcinoma); Caco-2 (colorectal adenocarcinoma); MDA-MB231 (breast adenocarcinoma); HCT116 (colorectal carcinoma); PC3 (prostatic adenocarcinoma); NCL-H727 (lung carcinoma); and MCF7 (breast cancer). After 24 h incubation with pseudonajide at 25 μM, there was no decrease in live cell counts compared to the control conditions (Fig. 10), indicating that pseudonajide is not cytotoxic to human cells.

## Discussion

Antimicrobial peptides are promising molecules in the fight against bacterial resistance [37]. Since AMPs can interact with a large variety of cell targets, they have an advantage in the fight against the production of bacterial resistance phenotypes [38]. Currently only three natural cathelicidins have had their antibiofilm activity reported (NA-CATH, CATHPb1, and SA-CATH) [7, 39–41]. Synthetic derivatives from these natural cathelicidins were tested and showed improved anti-biofilm activity in comparison with natural NA-CATH peptide [41].

Our first goal in the present work was to screen for new molecules with antibiofilm activity in two biofilm-forming models, *S. epidermidis* and *P. aeruginosa*. We have found that most of the 17 short snake-venom derived peptides tested here had no effect against *P. aeruginosa* (Additional file 8) or *S. epidermidis* (Additional file 9). We go on to demonstrate that an 11-amino acid peptide derived from *P. textilis* snake venom (peptide 2), herein named pseudonajide possesses antimicrobial activity against *S. epidermidis*. When we investigated the mechanism of action of pseudonajide, we found that it acts directly on the bacterial cell, and not on the biofilm matrix. This led us to investigate its antimicrobial activity, the molecule’s cellular binding site, as well as the bacterial molecules which might interact with this newly identified peptide.

To analyze the effects of pseudonajide on *S. epidermidis* cells, we performed growth curve and CFU experiments using a concentration of the peptide of 25 μM. We began by investigating the peptide’s effects in the early stages of interaction. In fact, it is possible to detect a great difference in the CFU counts after just 1, 2, and 4 h incubation, which is characteristic of a fast-acting antibiotic. Moreover, biofilm eradication activity was detected (Fig. 2d), with around 30% lower biofilm mass as compared to the control conditions, possibly due to the ability of pseudonajide to kill the biofilm-forming bacteria. This shows that the peptide has a dual action, both antimicrobial and against biofilm formation. To discover the binding sites of pseudonajide, we produced an FITC-tagged molecule. After 1, 2, and 24 h interaction with bacterial cells, confocal microscopy demonstrated that pseudonajide interacts with the *S. epidermidis* cell envelope (Fig. 4). We can therefore suggest that the first point of interaction for pseudonajide is the cell envelope, and not the biofilm matrix.

Based on their activities, AMPs can be divided into two main groups: they can act on the cell wall and disrupt the membrane, causing cell permeability; or they can have intracellular targets [42, 43]. Even though cationic peptides can have different amino acid sequences, they still have similar characteristics which permit interaction with bacterial cell membranes. As described in the literature, most of the residues in AMPs are positively charged and some are hydrophobic, ensuring the AMPs amphipathic character [31, 44]. In this work, structural analysis demonstrated that more than 50% of the amino acids which make up the peptide pseudonajide are positively charged (KRFKKFFMKLK). The presence of methionine (M) or suppression of lysine (K) at position 8 seems to increase the antimicrobial/antibiofilm activity of pseudonajide. Methionine residue was not seen in peptides 1 (KRFKKFFKKVK) or 3 (KRFKKFFKKLK).The antimicrobial peptide sequence from which pseudonajide was identified and synthesized was originally reported by Falcão’s group [10], and belongs to the vipericidins, a family of cathelicidin-related peptides derived from the venom glands of South American pit vipers. They described these vipericidins as having antimicrobial activities against different bacteria, including *S. aureus* and *P. aeruginosa* strains. We have evidence that pseudonajide is acting on the cell wall and membrane of *S. epidermidis.* It is reported in the literature that AMPs bind preferentially to the cationic bacterial membrane instead of the zwitterionic membrane in mammalian cells [44]. Moreover, pseudonajide contains 36% hydrophobic amino acids, a characteristic which may explain its interactions with the bacterial cell membrane. Insertion of the peptide into the hydrophobic portion of the membrane seems to cause osmotic imbalance in the cell, which could lead to the shrunken cell morphology observed in the SEM (Fig. 6) and TEM (Fig. 7) analyses. It is important to note that in TEM, the defective cells have external material surrounding them. We hypothesize that this consists of extravasated DNA and disorganized peptidoglycan, but more tests are necessary to prove it. We also surmise that the smaller cells that can be observed are the same as those seen on FITC-tagged peptides with confocal microscopy. The green fluorescent cell sizes were all smaller than those of the non-fluorescent cells. In summary, pseudonajide acts on the cell envelope, likely inducing osmotic stress that leads to a reduction in cell size (Fig. 5).

**Fig. 11:**
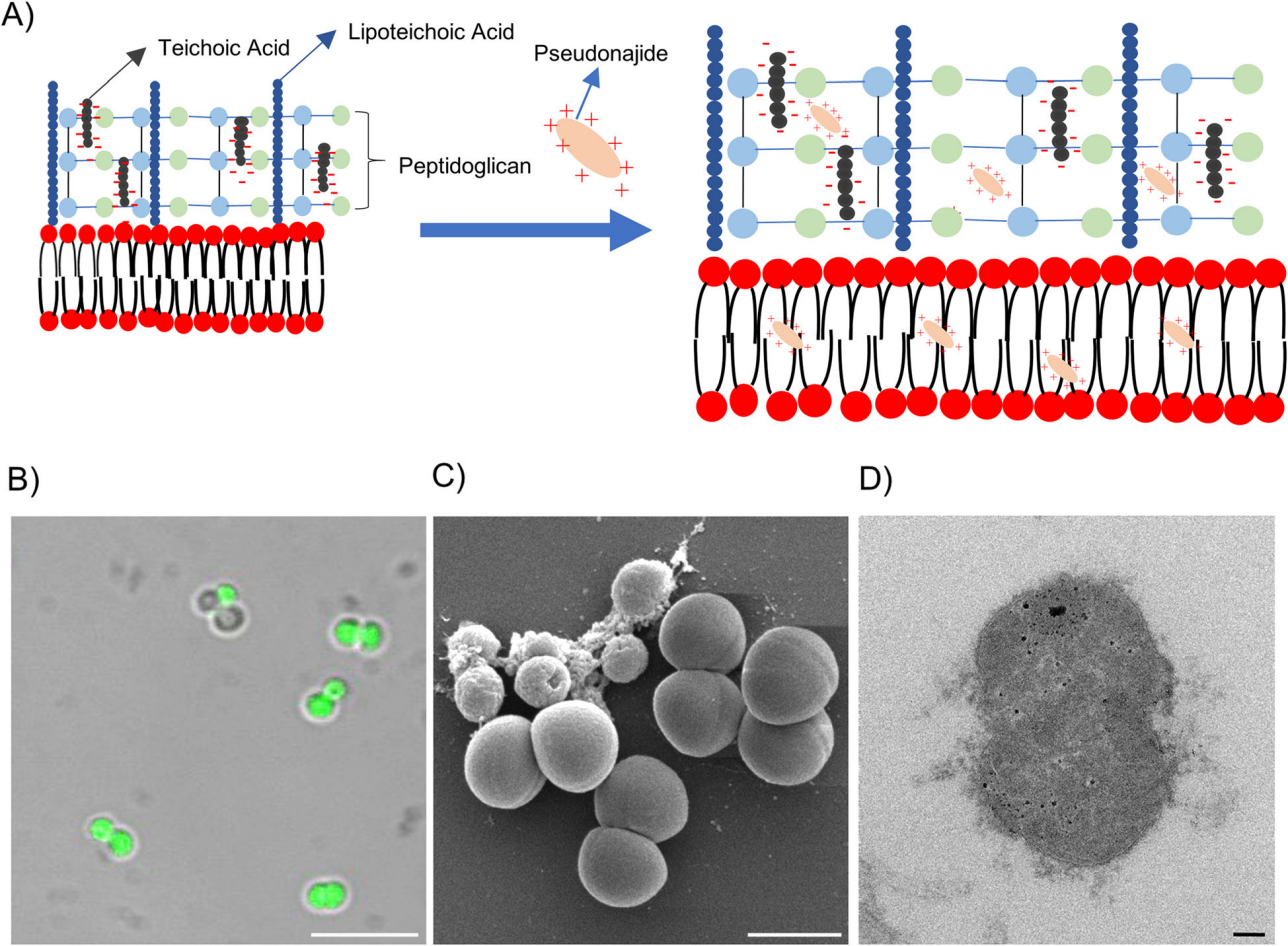
Proposed mechanism of action for pseudonajide (peptide 2). **a** Schematic of the proposed mechanism of action of pseudonajide in the cell walls and membranes of Gram-positive bacteria. The cationic peptide is shown interacting with negative charges from the cell wall components and with the hydrophobic portions of bacterial membranes. Such interactions lead to: (**b**) cell envelope binding, shown in a confocal microscopy image; (**c**) defective morphology, observed by scanning electron microscopy; and (**d**) cell wall and membrane fractures, observed by transmission electron microscopy

The cell wall in Gram-positive bacteria is a complex network of molecules in a structure composed mainly of peptidoglycan and teichoic acids. Teichoic acids are negatively charged poly-glycerophosphate chains that can be linked to peptidoglycan or anchored to the cytoplasmic membrane [45, 46]. Moreover, D-alanylation of lipoteichoic acid is said to promote protection against cationic AMPs in Gram-positive bacteria [47]. In order to test this, we assessed the expression levels of genes coding for LTA assembly molecules, namely glycosyltransferase YgfP (UgtP), flippase LtaA, and lipoteichoic acid synthase LtaS [48]. In *S. aureus,* lipoteichoic acid synthesis starts with YgfP, encoded by the *ugtP* gene. This protein synthesizes the glycolipid anchor Glc2-DAG from UDP-Glc and diacylglycerol (DAG). Glc2-DAG is translocated to the outside of the membrane by LtaA [48, 49], and elongation of the LTA chain is then promoted by LtaS [48, 50]. Based on the literature and due to the physicochemical characteristics of teichoic acids, we speculated that pseudonajide must act on teichoic acids on *S. epidermidis* cell wall. We detected increased expression of all three tested genes when the cells were cultured in the presence of pseudonajide (Fig. 8b). Our fluorescence polarization experiments indicated an interaction between pseudonajide and lipoteichoid acids, which probably caused cell wall disorganization in *S. epidermidis* (Fig. 11). The induced increase in gene expression may well be a compensatory mechanism to protect against the presence of the peptide, preserving cell viability. It was already described that exposure to daptomycin, a lipopeptide antibiotic, causes increased expression of lipoteichoic acid-related genes, and that is associated with cell wall stress [47, 51].

It was previously suggested that cationic antimicrobial peptides kill bacterial cells. They first interact with the membrane through electrostatic interactions [52], contacts which result in membrane disruption and cell death. Other peptides can cross the bacterial lipid bilayer without causing any damage to the cell membrane, but still inhibit intracellular functions, so they also eventually lead to bacterial death. Pseudonajide consists of an amino acid sequence (KRFKKFFMKLK) that is part of a peptide isolated from *P. textilis* venom. Among the peptides we tested, pseudonajide has the best anti biofilm formation activity, at a sub-MIC concentration of just 6.25 uM (Fig. 2b), and the best eradication of established biofilm activity in the group (Fig. 2d). Several AMPs have been described as also having antimicrobial activity against Gram-negative bacteria. In the present work, we did not observe any antibiofilm or antimicrobial activity against *P. aeruginosa* (Additional file 8). Further studies are needed to assess possible mechanisms that explain this overall lack of activity against *P. aeruginosa*. Short peptides such as the ones we tested might suffer degradation by *P. aeruginosa* proteases [53, 54], since small peptides are typically more susceptible to these enzymes. A *P. aeruginosa* metalloprotease is probably involved in degradation of an antimicrobial peptide named GL13K [55]. In *Proteus mirabilis*, the ZapA-zinc metalloprotease is able to cleave LL-37 as well as β-defensin 1 [56]. The inhibition of LL-37 bactericidal activity by alginate and exopolysaccharides is another example of antimicrobial peptide defense reported in *P. aeruginosa*. The inhibition occurs through LL-37 sequestration, which diminishes AMP concentrations at the target site [57].

We have observed pseudonajide’s dual activity, as it is both antimicrobial and also inhibiting *S. epidermidis* biofilm formation*.* Even though we did not see any alteration in the expression of biofilm-related genes when the peptide was present, we did observe biofilm eradication with reduction in mass (Fig. 2b). This decrease can be explained by several elements. One is the relationship between the cell wall teichoic and lipoteichoic acids and the processes of adhesion and biofilm formation [58]. In *S. epidermidis,* cell wall teichoic acids seem to induce adhesion-immobilized fibronectin [59]. Moreover, these types of molecules have been detected in the biofilm matrix of *S. epidermidis* [60]. If pseudonajide mainly acts on teichoic and lipoteichoic acids, the reduction in adhesion could be one of the causes of both biofilm reduction and outright eradication. It is also important to emphasize the characteristics of cationic antibiofilm peptides, described by Von Borowski et al. [61]. They showed that lysine (K) and phenylalanine (F) are the most frequently found amino acids in antibiofilm peptides, and this is clearly also the case for pseudonajide (KRFKKFFMKLK).

## Conclusions

We have showcased here the promising activity of a synthetic peptide derived from *P. textilis* venom. Its dual action against *S. epidermidis* cells and biofilms make pseudonajide a very promising molecule for new drug development, and this is reinforced by the fact that it has a short 11 amino acid sequence. Shorter sequences are advantageous both for industry and antimicrobial peptide researchers, as they are easier to synthesize and cost less. Importantly, this facilitates future research into their structures and into ways to improve their efficiency.

## Methods

### Peptides alignment and synthesis

A list of 170 antimicrobial peptides from animal venoms, reviewed previously by our research group [1], was used to perform amino acid sequence alignments in Clustal X, to investigate the presence of conserved motifs and to gain structural and functional insights about those AMP sequences. Alignments were analyzed and the selected sequences were synthesized by GenOne Biotechnologies®, with respective HPLC and mass spectrum analysis (Additional files 10, 11, 12, 13, 14, 15, 16, 17, 18, 19, 20, 21, 22, 23, 24, 25 and 26). The synthetic peptides were then solubilized in 0.5% DMSO and used for further testing.

### Bacterial strains and culture conditions

*S. epidermidis* ATCC 35984 and *Pseudomonas aeruginosa* PAO1 were used to test the antimicrobial and antibiofilm activities of the peptides. Both bacteria were grown in blood agar plates and cultured overnight at 37 °C. Cell suspensions were prepared in a solution of 0.9% NaCl and adjusted to OD_600_ for a final concentration of _~_ 10^8^ cells/ml. For microscopy analysis, pre-inoculum was made in tryptic soy broth (TSB, Merck), and adjusted to OD_600_ for the same concentration of cells for all tests.

### Tests of antimicrobial and antibiofilm activity

Serial dilution of peptides was performed in 96-well plates, going from 100 to 3.12 μM. Cell suspensions and TSB were added to the plates and a control was made with dimethyl sulfoxide (DMSO). Antibiofilm formation tests were performed with an adapted protocol [62], in which it is described that 24 h of incubation is sufficient to determinate antibiofilm activity. The OD_600_ was measured, then biofilm content accessed using the crystal violet protocol [29]. Biofilm eradication test was performed supplementing 24 h pre-formed biofilm with a new culture broth, containing or not 25 μM of peptide. The plates were incubated for further 24 h followed by crystal violet protocol. The antimicrobial activity of pseudonajide was analyzed using a concentration of 25 μM after 1, 2, 4, and 24 h incubation. After measuring the optical density, the supernatant was collected and diluted. A volume of 100 μl was plated in Luria broth agar plates. CFUs were counted after 24 h incubation. All experiments were performed at least three different times, each with three technical replicates.

### Scanning electron microscopy

*S. epidermidis* was cultured in the same conditions as described previously, in the presence or absence of 25 μM pseudonajide. However, for this analysis, a plastic slide was placed inside each well, and these plates were incubated for 1, 4, and 24 h. After incubation, the plastic slides were washed three times with 0.9% NaCl solution and fixed overnight in fixation buffer (2.5% glutaraldehyde, 2% paraformaldehyde, 0.1 M sodium cacodylate) at 4 °C. The adhered cells were then dehydrated with increasing concentrations of ethanol solutions. The images were obtained using a JSM-7100F scanning electron microscope (JEOL).

### Transmission electron microscopy

Bacterial cells were cultured in 24-well plates in the presence or absence of 25 μM of pseudonajide. Cells were incubated for 1, 4, and 24 h. All of the content in the well was recovered, centrifuged at 12,000 g, then washed with saline solution. Fixation was performed for 18 h at 4 °C in buffer (0.2 M sodium cacodylate, 16% paraformaldehyde, 25% glutaraldehyde, 75 mM lysine). Samples were then washed 4 times with a solution containing 0.1 M sodium cacodylate and 0.2 M sucrose. After each resuspension, samples were incubated for 10 min in this solution. Contrast was done for 1 h with a solution of 1% osmium tetroxide and 1.5% potassium ferrocyanure. Dehydration was induced by gradually introducing a solution of ethanol, and infiltration with increasing concentrations of LR white resin (Delta Microscopies) diluted in ethanol. Inclusion and polymerization were performed over 24 h at 60 °C in capsules with LR white resin in the absence of O_2_. Thin sections (80 nm) were collected onto 200-mesh carbon grids and visualized with a FEI Tecnai Sphera microscope operating at 200 kV and equipped with a Gatan 4x4k UltraScan CCD camera.

### Confocal microscopy

Bacterial cultures were done in the same conditions as described above, but they were incubated with pseudonajide tagged with FITC. After incubation, cells were washed with saline solution, then 3 μL were added to glass slides for confocal analysis. The images were acquired using a Leica SP8 DMI 6000 CS confocal microscope, and ImageJ software was used for image analysis.

### Gene expression analysis

Quantitative real-time PCR (qRT-PCR) was performed on RNA extracted from bacteria cultured during 4 h with or without 6.25 μM of pseudonajide. Nucleic acids were extracted using TRIzol reagent (Thermo Fisher) following the manufacturer’s protocol. RNAse-free DNAse I (NEB) was added to 2 μg RNA, then 1 μg RNA was used for reverse transcriptase (RT) reactions with M-MLV reverse transcriptase enzymes (Promega). RT reactions were done using Random Primers (Promega). For the qRT-PCR assay, 10 ng cDNA was used with SYBR Green PCR Master Mix (Applied Biosystems) supplemented with the respective primers [48]. The reactions were performed in a StepOne Real-Time system (Thermo Fisher). Expression levels of the 16S rRNA gene were used for the relative gene expression normalization analysis [34]. The primers used in this work are listed in Table 1.

**Table 1:** Primers used in this work

Gene	Forward primer 5'-3'	Reverse primer 5'-3'	Amplicon
*atlE*	TACCAGGGTTTGCAGGATTC	GGCGCTAAATTCATTGGAAA	85 bp
*aap*	AGGCCGTACCAACAGTGAAT	ATGGGCAAACGTAGACAAGG	100 bp
*agrC*	TCATCAATATCGCATTCATCG	CCTAAACCGCGATTATCACC	136 bp
*icaA*	TTATCAATGCCGCAGTTGTC	CCGTTGGATATTGCCTCTGT	104 bp
*leuA*	GATGATCTCGGAATGGCAGT	TGAGGCATTTCCTGCTCTTT	108 bp
*saeR*	GCTAACACTGTCAATGTCCACA	AGGCCCCACACAGTTGTAAT	92 bp
*saeS*	GGCGTCAATTTGTTGTGCTA	AGGGCATAGGTATCGTTCCA	140 bp
*sarA*	TTTGCTTCTGTGATACGGTTGT	CGTAATGAACACGATGAAAGAACT	107 bp
*ugtP* (45)	AAGCTGGTGTTCCTGCTTC	ATGACCACTTGCGAATTTGG	216 bp
*ltaA* (45)	GCCTTGGTTGTGCTTATTGC	GGAAGAATAGGTACAAGTGC	184 bp
*ltaS* (45)	ATGGTAAAGAGGTTACACC	CCTTCAGTGAATAGGCTGAG	172 bp
*qPCR16sRNA*(45)	AGGAGTCTGGACCGTGTCTC	GCGTAGCCGACCTGAGAG	51 bp

### Toxicity test

Cytotoxicity tests were performed on the ImPACcell robotic platform (BIOSIT, Université de Rennes 1). This featured high-throughput multiparameter image analysis, with both high-content screening and high-content analysis. The platform is equipped with an Olympus microscope and Compix SimplePCI software; a Zeiss Axio Imager M1 microscope with a Zeiss camera and AxioVision software; and imaging systems including an ArrayScan VTI Cellomics reader (Thermo Fisher), Hamilton STARlet and NIMBUS workstations, and a Scienion spotter. Cells used in the test were obtained from an already-existing collection available at BIOSIT (https://biosit.univ-rennes1.fr/impaccell-imagerie-pour-analyse-du-contenu-cellulaire). For the tests, seven different cell lines were used: human hepatocellular carcinoma (HuH7); colorectal adenocarcinoma (Caco-2); breast adenocarcinoma (MDA-MB231); colorectal carcinoma (HCT116); prostatic adenocarcinoma (PC3); lung carcinoma (NCL-H727); and breast cancer (MCF7). The residual cell percentages reported correspond to viable cells compared to the average viable cells in the DMSO control. Viability of 100% represents no cytotoxicity or inhibition of cell growth, while under 25–30% is considered cytotoxic and 0% represents acute cytotoxicity.

### Fluorescence polarization

Fluorescence polarization measurements were conducted on a CLARIOstar (BMG Labtech) spectrophotometer in a Greiner 364-well flat bottom black microplate. Data were acquired at 23 °C in water using the FITC fluorescence of FITC-pseudonajide and 35 flashes in standard mode. FITC-pseudonajide concentration was set to 5 μM and volume was adjusted to 20 μL in all wells. *S. aureus* LTA was purchased from Sigma. Titration series was set in triplicate using LTA monomer concentrations ranging from 2.5 μM to 5 mM. Data were recorded within one hour, analyzed using Kaleidagraph (Synergy Software) and fitted using a 1:1 model and the following equation: P_obs_ = P_free_ + [[(P_max_-P_free_)/2C_0_] * [(K_d_ + L_0_ + C_0_)-((K_d_ + L_0_ + C_0_)^^2^-4C_0_L_0_)^^0.5^]] with P = polarization, C_0_ = pseudonajide concentration, L_0_ = total LTA concentration.

## Supplementary information

**Additional file 1.** Alignment of ant peptide sequences using Clustal X program.

**Additional file 2.** Alignment of bee peptide sequences using Clustal X program.

**Additional file 3.** Alignment of centipede peptide sequences using Clustal X program.

**Additional file 4.** Alignment of cone snail peptide sequences using Clustal X program.

**Additional file 5.** Alignment of scorpion peptide sequences using Clustal X program.

**Additional file 6.** Alignment of spider peptide sequences using Clustal X program.

**Additional file 7.** Alignment of wasp peptide sequences using Clustal X program.

**Additional file 8 **Antibiofilm formation testing of 16 short peptides in *P. aeruginosa*. Graphs demonstrating biofilm mass quantification at OD_570_ by the crystal violet protocol (left) and growth at OD_600_ (right) in the presence of different concentrations of 16 peptides. Tests were performed over 24 h. Gentamicin was used as the antibiofilm and antibiotic control, while TSB culture medium and TSB containing 2% DMSO were used as biofilm formation and growth controls.

**Additional file 9 **Antibiofilm formation activity screening of 14 short peptides in *S. epidermidis*. Graphs showing biofilm mass quantification at an optical density of 570 nm (OD_570_) using the crystal violet protocol (light gray), and growth at OD_600_ (dark gray), in the presence of different concentrations of 14 peptides. TSB culture medium with 2% dimethyl sulfoxide (DMSO) was used as a control for biofilm formation and growth, and rifampicin was the antibiofilm and antibiotic positive control. OD_600_ was measured at time zero and at 24 h for growth normalization.

**Additional file 10.** Peptide 1: A) HPLC analysis chromatogram. B) Mass spectrum.

**Additional file 11.** Peptide 2: A) HPLC analysis chromatogram. B) Mass spectrum.

**Additional file 12.** Peptide 3: A) HPLC analysis chromatogram. B) Mass spectrum.

**Additional file 13.** Peptide 4: A) HPLC analysis chromatogram. B) Mass spectrum.

**Additional file 14.** Peptide 5: A) HPLC analysis chromatogram. B) Mass spectrum.

**Additional file 15.** Peptide 6: A) HPLC analysis chromatogram. B) Mass spectrum.

**Additional file 16.** Peptide 7: A) HPLC analysis chromatogram. B) Mass spectrum.

**Additional file 17.** Peptide 8: A) HPLC analysis chromatogram. B) Mass spectrum.

**Additional file 18.** Peptide 9: A) HPLC analysis chromatogram. B) Mass spectrum.

**Additional file 19.** Peptide 10: A) HPLC analysis chromatogram. B) Mass spectrum.

**Additional file 20.** Peptide 11: A) HPLC analysis chromatogram. B) Mass spectrum.

**Additional file 21.** Peptide 12: A) HPLC analysis chromatogram. B) Mass spectrum.

**Additional file 22.** Peptide 13: A) HPLC analysis chromatogram. B) Mass spectrum.

**Additional file 23.** Peptide 14: A) HPLC analysis chromatogram. B) Mass spectrum.

**Additional file 24.** Peptide 15: A) HPLC analysis chromatogram. B) Mass spectrum.

**Additional file 25.** Peptide 16: A) HPLC analysis chromatogram. B) Mass spectrum.

**Additional file 26.** Peptide 17: A) HPLC analysis chromatogram. B) Mass spectrum.

## Data Availability

The datasets used and/or analyzed during the current study are available from the corresponding author on reasonable request.

## Abbreviations

AMPs: Antimicrobial peptides
APD: Antimicrobial Peptide Database
CFU: Colony-forming units
DAG: Diacylglycerol
DMSO: Dimethyl sulfoxide
FITC: Fluorescein isothiocyanate
FP: Fluorescence polarization
LTA: Lipoteichoic acid
qRT-PCR: Quantitative real-time PCR
RT: Reverse transcriptase
SEM: Scanning electron microscopy
TEM: Transmission electron microscopy
TSB: Tryptic soy broth
