# Supplementary figures and images for: Pseudonajide peptide derived from snake venom alters cell envelope integrity interfering on biofilm formation in Staphylococcus epidermidis
Source: BMC Microbiol. 2020 Aug 3;20:237. doi: 10.1186/s12866-020-01921-5 (PMC7397659; doi:10.1186/s12866-020-01921-5)

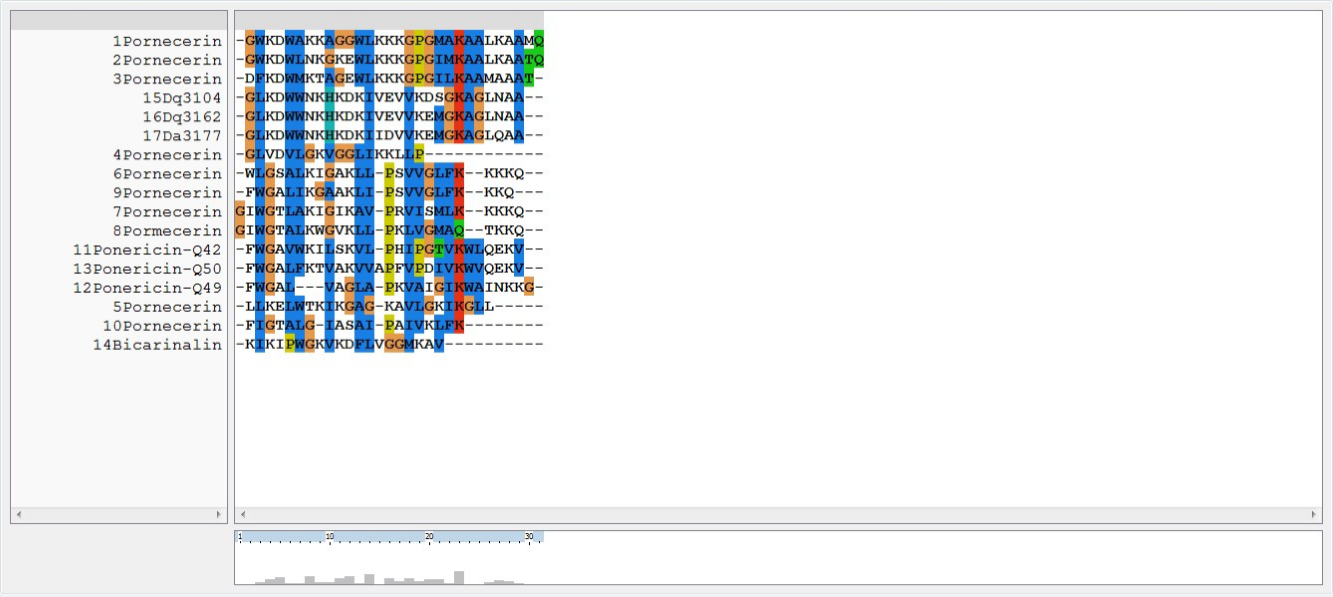

Supplement: Supplementary file 1 — Additional file 1. Alignment of ant peptide sequences using Clustal X program. [file 12866_2020_1921_MOESM1_ESM.tif]

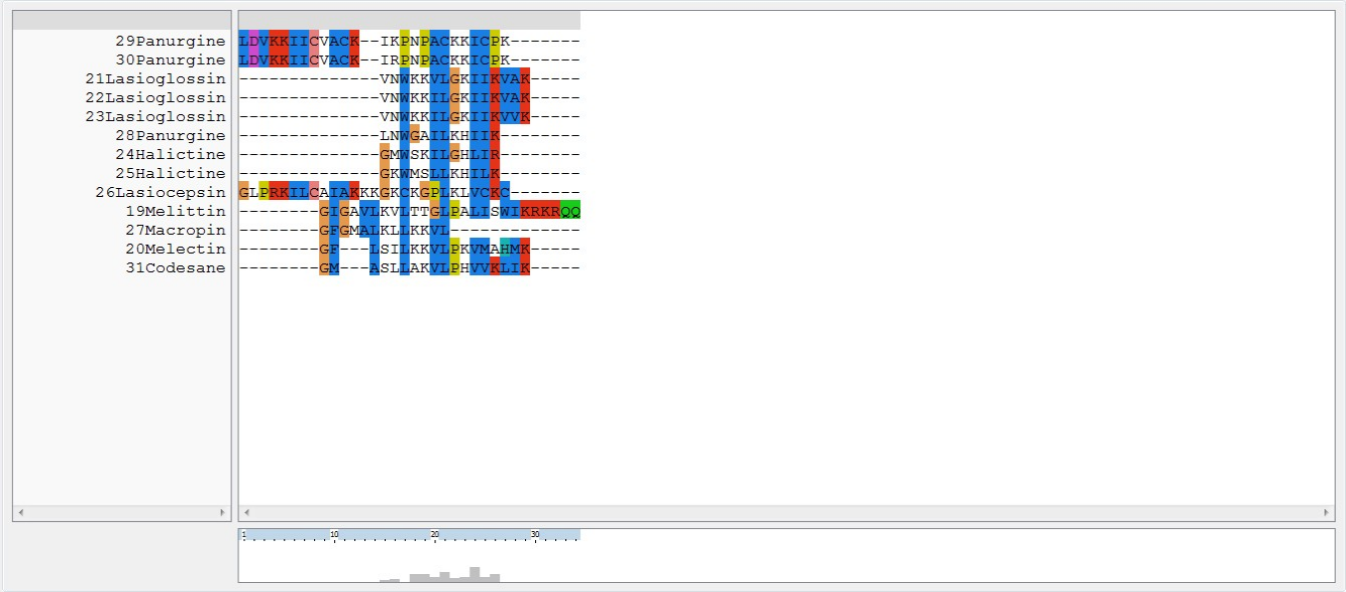

Supplement: Supplementary file 2 — Additional file 2. Alignment of bee peptide sequences using Clustal X program. [file 12866_2020_1921_MOESM2_ESM.tif]

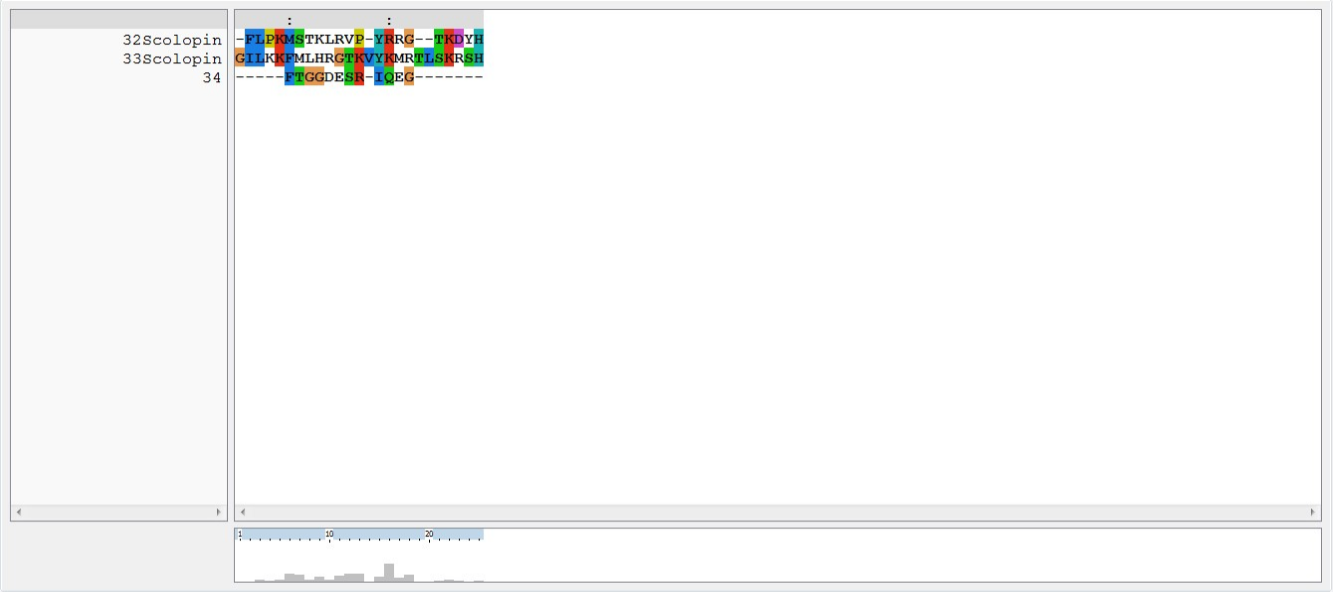

Supplement: Supplementary file 3 — Additional file 3. Alignment of centipede peptide sequences using Clustal X program. [file 12866_2020_1921_MOESM3_ESM.tif]

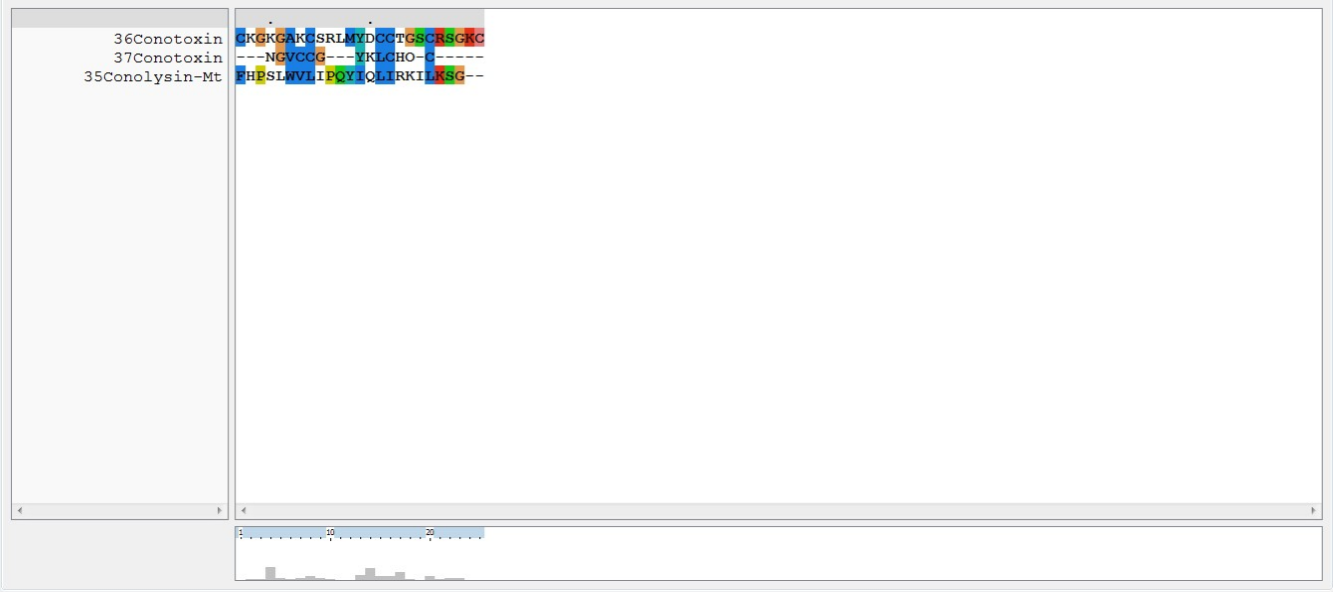

Supplement: Supplementary file 4 — Additional file 4. Alignment of cone snail peptide sequences using Clustal X program. [file 12866_2020_1921_MOESM4_ESM.tif]

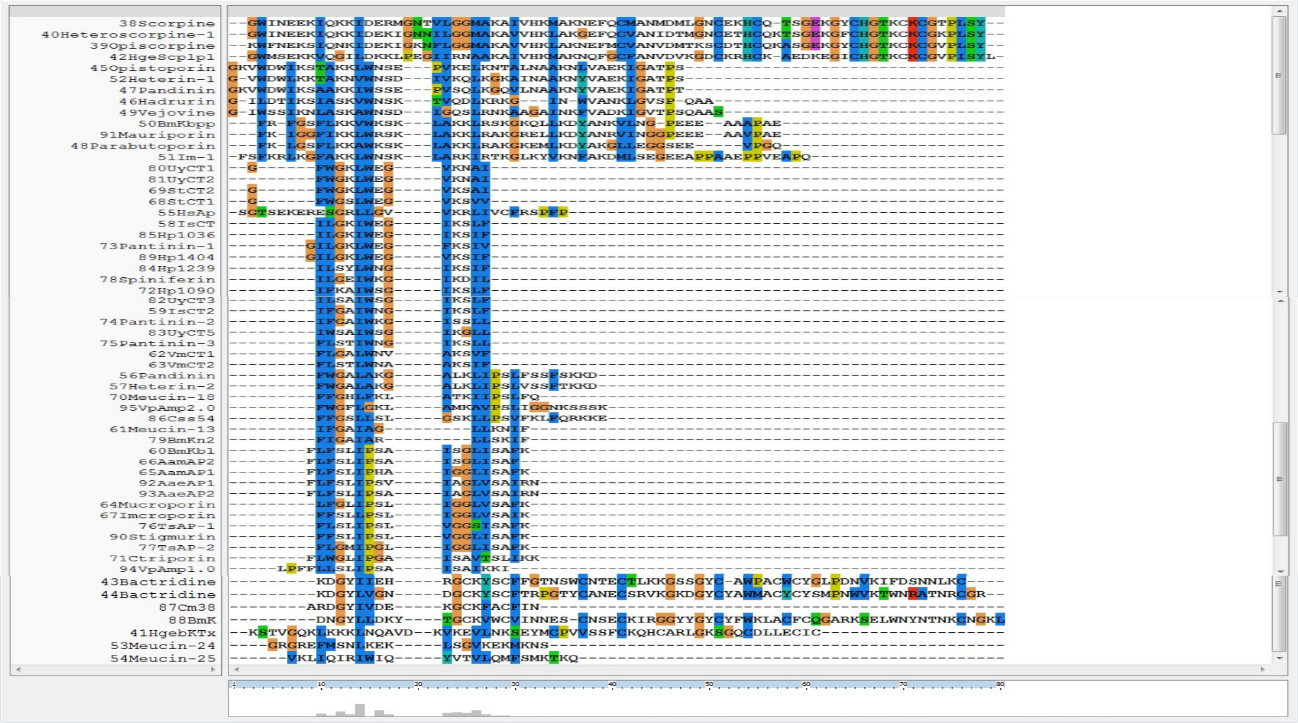

Supplement: Supplementary file 5 — Additional file 5. Alignment of scorpion peptide sequences using Clustal X program. [file 12866_2020_1921_MOESM5_ESM.tif]

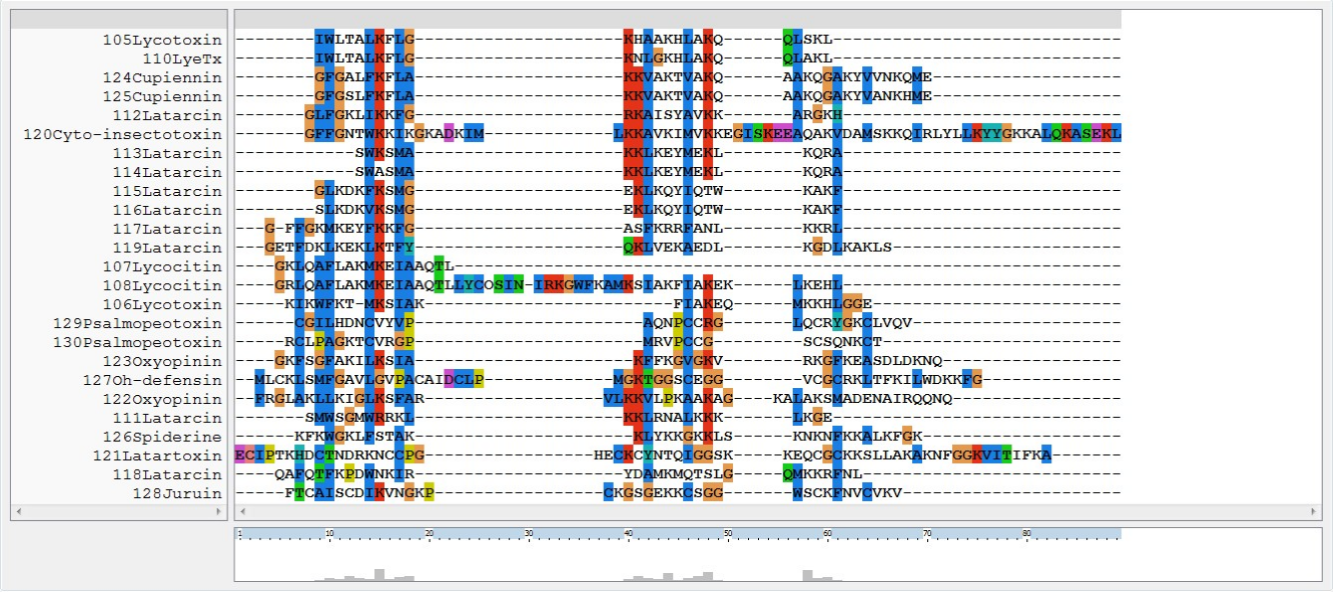

Supplement: Supplementary file 6 — Additional file 6. Alignment of spider peptide sequences using Clustal X program. [file 12866_2020_1921_MOESM6_ESM.tif]

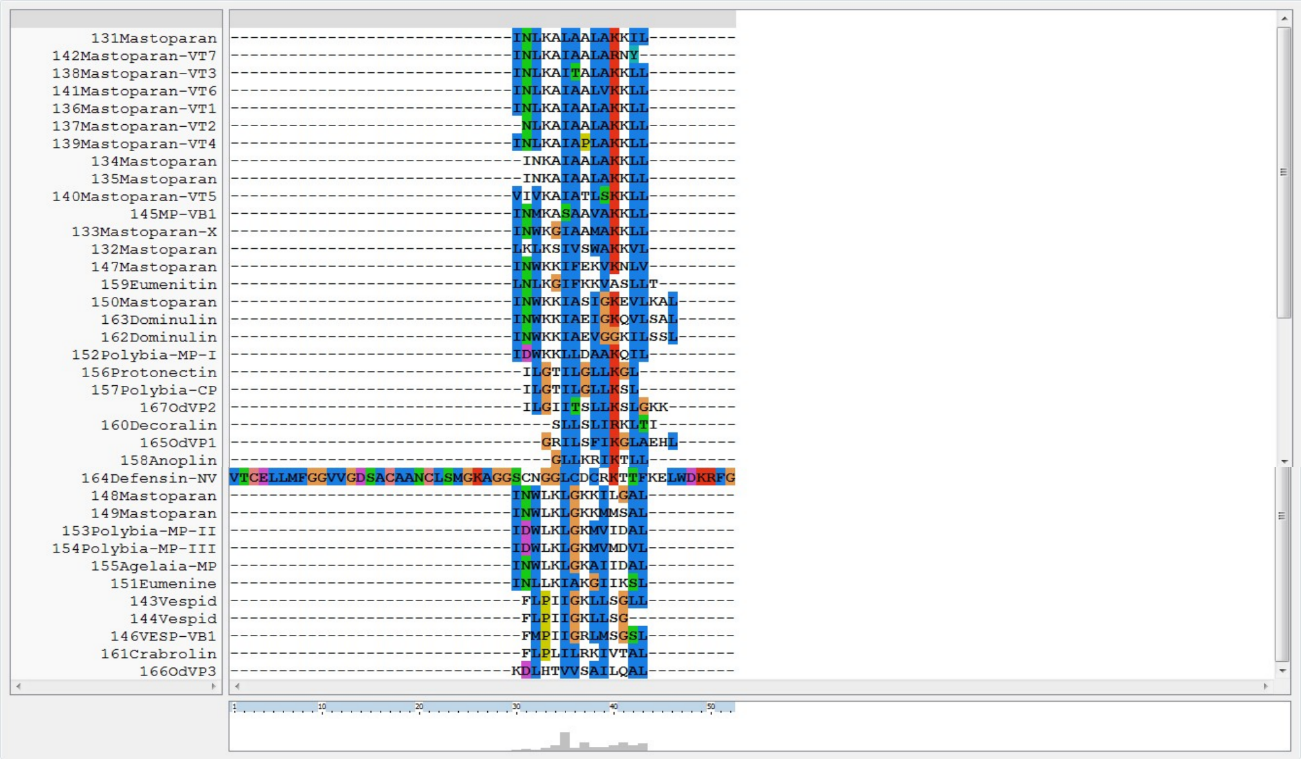

Supplement: Supplementary file 7 — Additional file 7. Alignment of wasp peptide sequences using Clustal X program. [file 12866_2020_1921_MOESM7_ESM.tif]

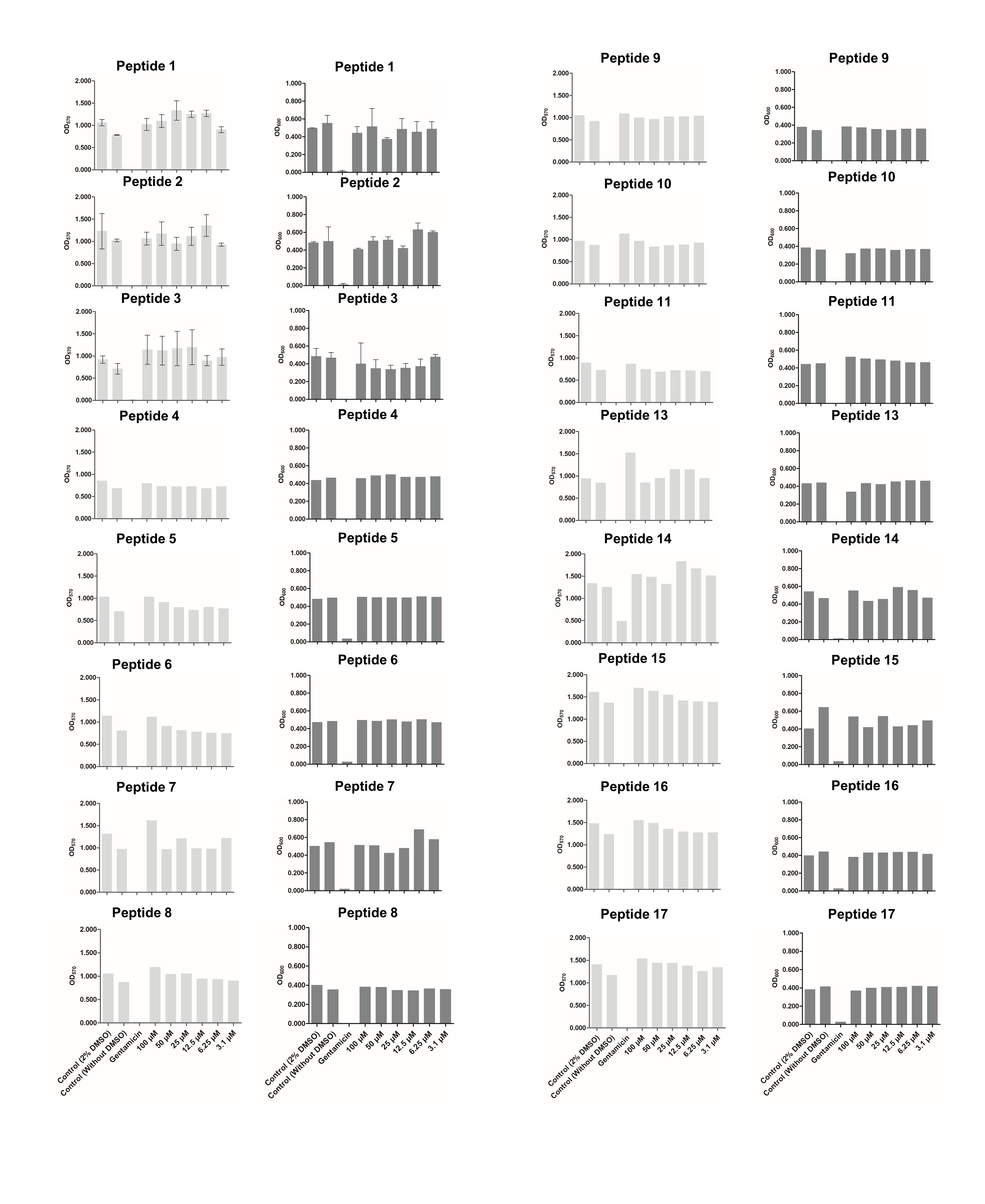

Supplement: Supplementary file 8 — Additional file 8 Antibiofilm formation testing of 16 short peptides in P. aeruginosa. Graphs demonstrating biofilm mass quantification at OD570 by the crystal violet protocol (left) and growth at OD600 (right) in the presence of different concentrations of 16 peptides. Tests were performed over 24 h. Gentamicin was used as the antibiofilm and antibiotic control, while TSB culture medium and TSB containing 2% DMSO were used as biofilm formation and growth controls. [file 12866_2020_1921_MOESM8_ESM.tif]

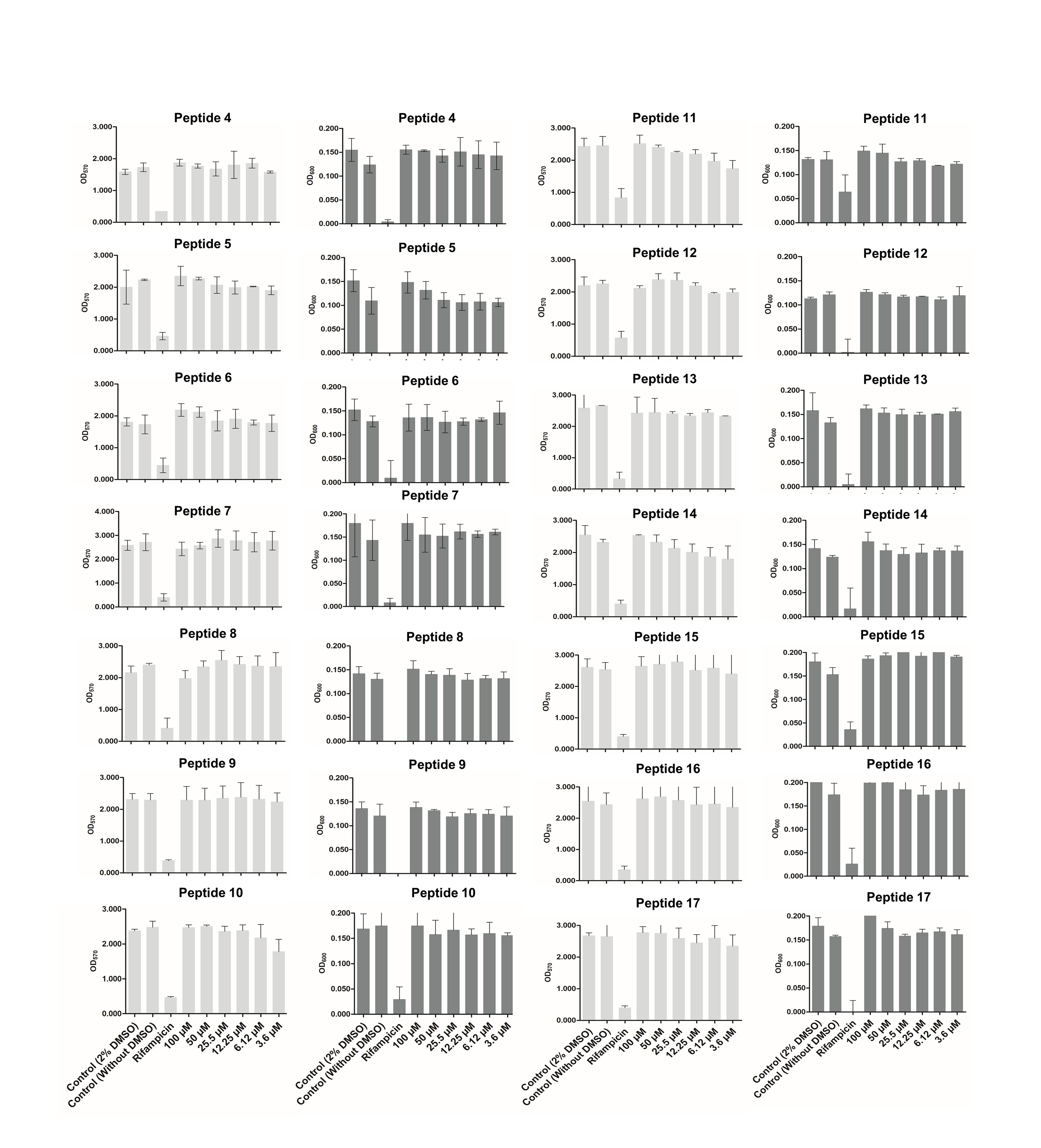

Supplement: Supplementary file 9 — Additional file 9 Antibiofilm formation activity screening of 14 short peptides in S. epidermidis. Graphs showing biofilm mass quantification at an optical density of 570 nm (OD570) using the crystal violet protocol (light gray), and growth at OD600 (dark gray), in the presence of different concentrations of 14 peptides. TSB culture medium with 2% dimethyl sulfoxide (DMSO) was used as a control for biofilm formation and growth, and rifampicin was the antibiofilm and antibiotic positive control. OD600 was measured at time zero and at 24 h for growth normalization. [file 12866_2020_1921_MOESM9_ESM.tif]

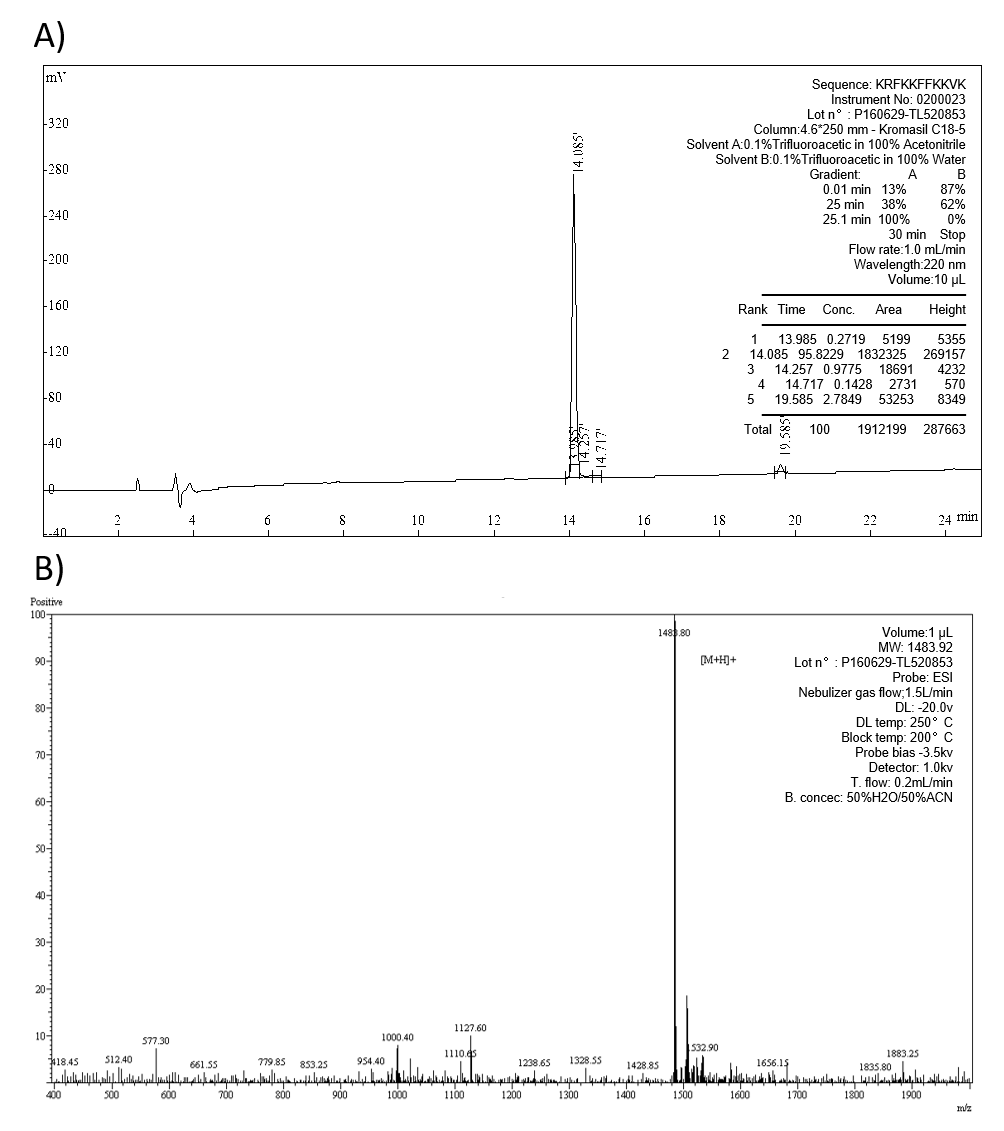

Supplement: Supplementary file 10 — Additional file 10. Peptide 1: A) HPLC analysis chromatogram. B) Mass spectrum. [file 12866_2020_1921_MOESM10_ESM.tif]

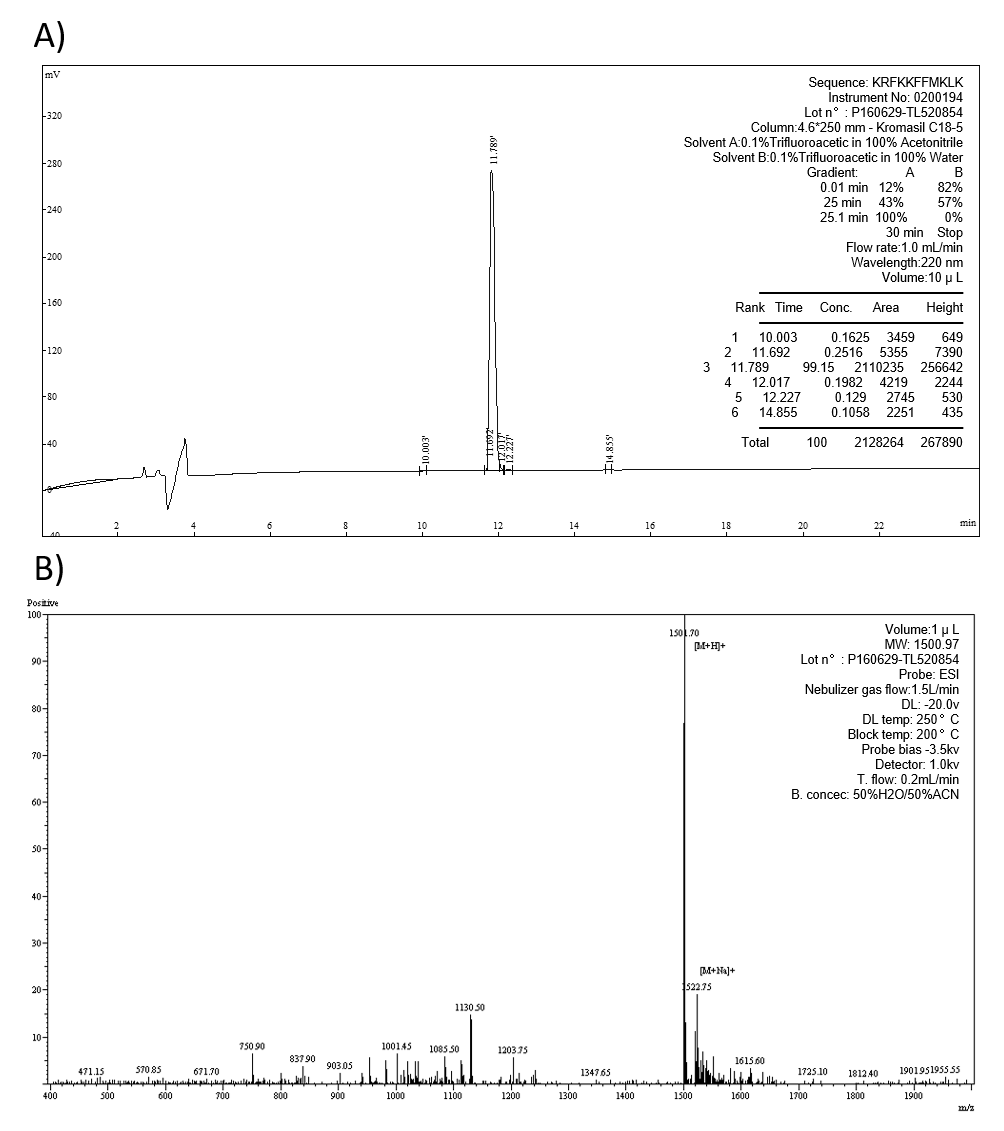

Supplement: Supplementary file 11 — Additional file 11. Peptide 2: A) HPLC analysis chromatogram. B) Mass spectrum. [file 12866_2020_1921_MOESM11_ESM.tif]

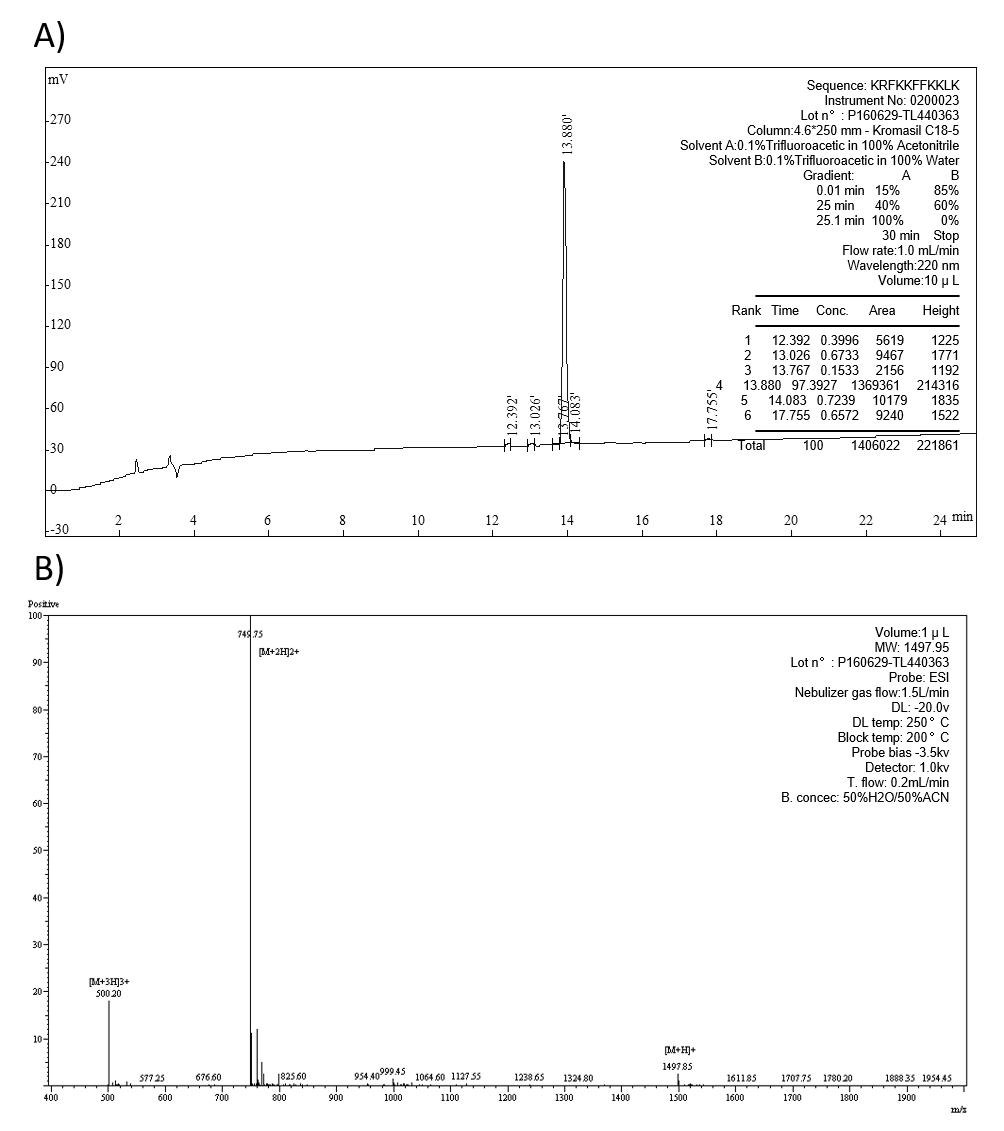

Supplement: Supplementary file 12 — Additional file 12. Peptide 3: A) HPLC analysis chromatogram. B) Mass spectrum. [file 12866_2020_1921_MOESM12_ESM.tif]

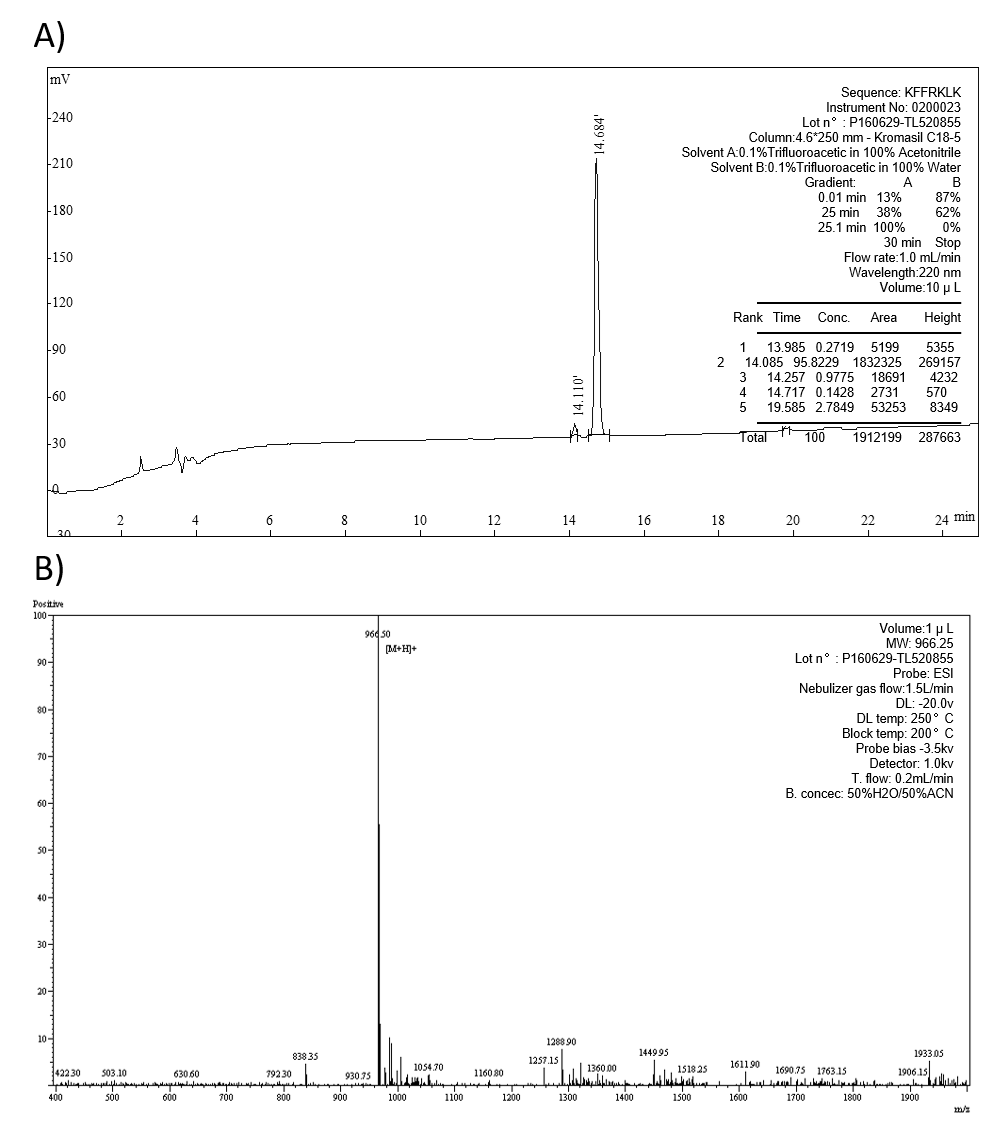

Supplement: Supplementary file 13 — Additional file 13. Peptide 4: A) HPLC analysis chromatogram. B) Mass spectrum. [file 12866_2020_1921_MOESM13_ESM.tif]

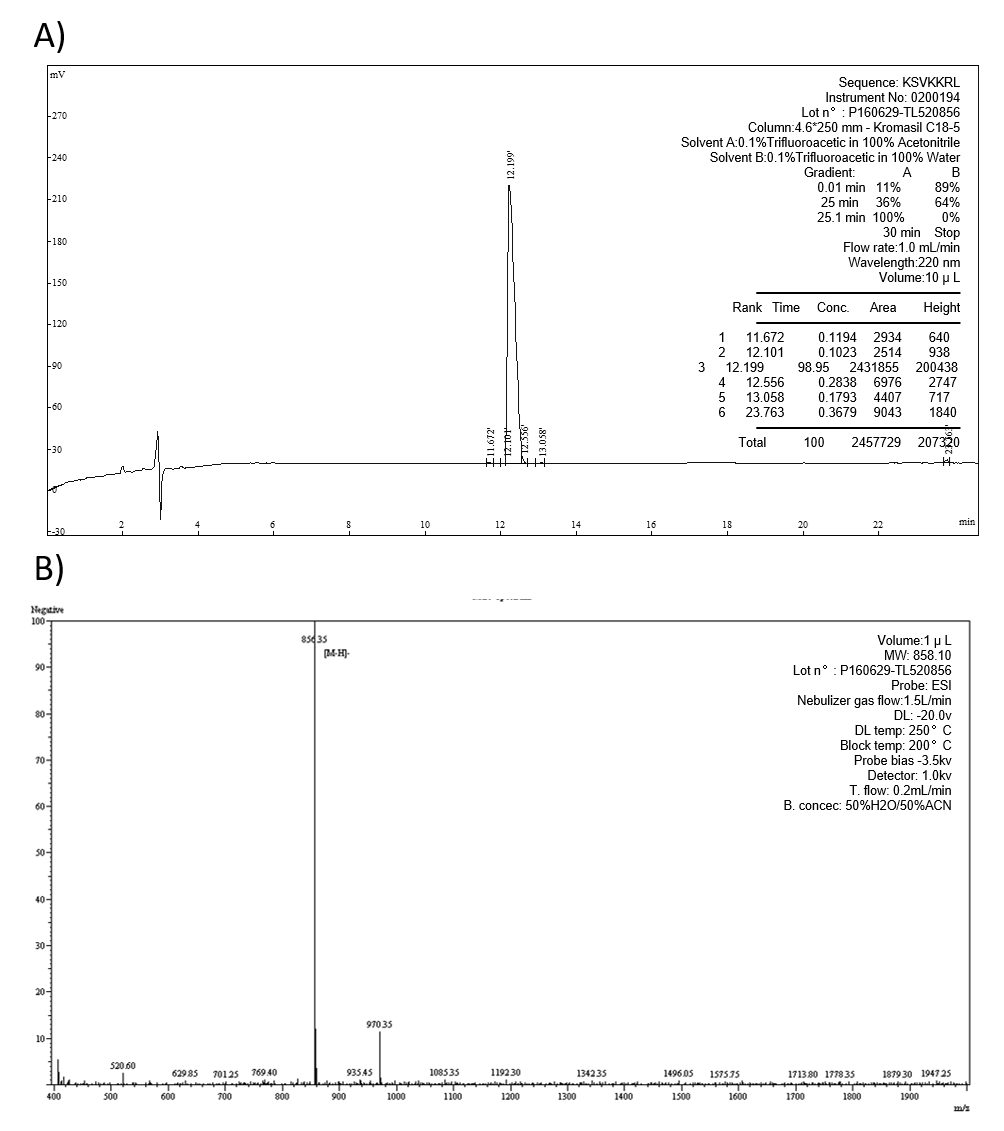

Supplement: Supplementary file 14 — Additional file 14. Peptide 5: A) HPLC analysis chromatogram. B) Mass spectrum. [file 12866_2020_1921_MOESM14_ESM.tif]

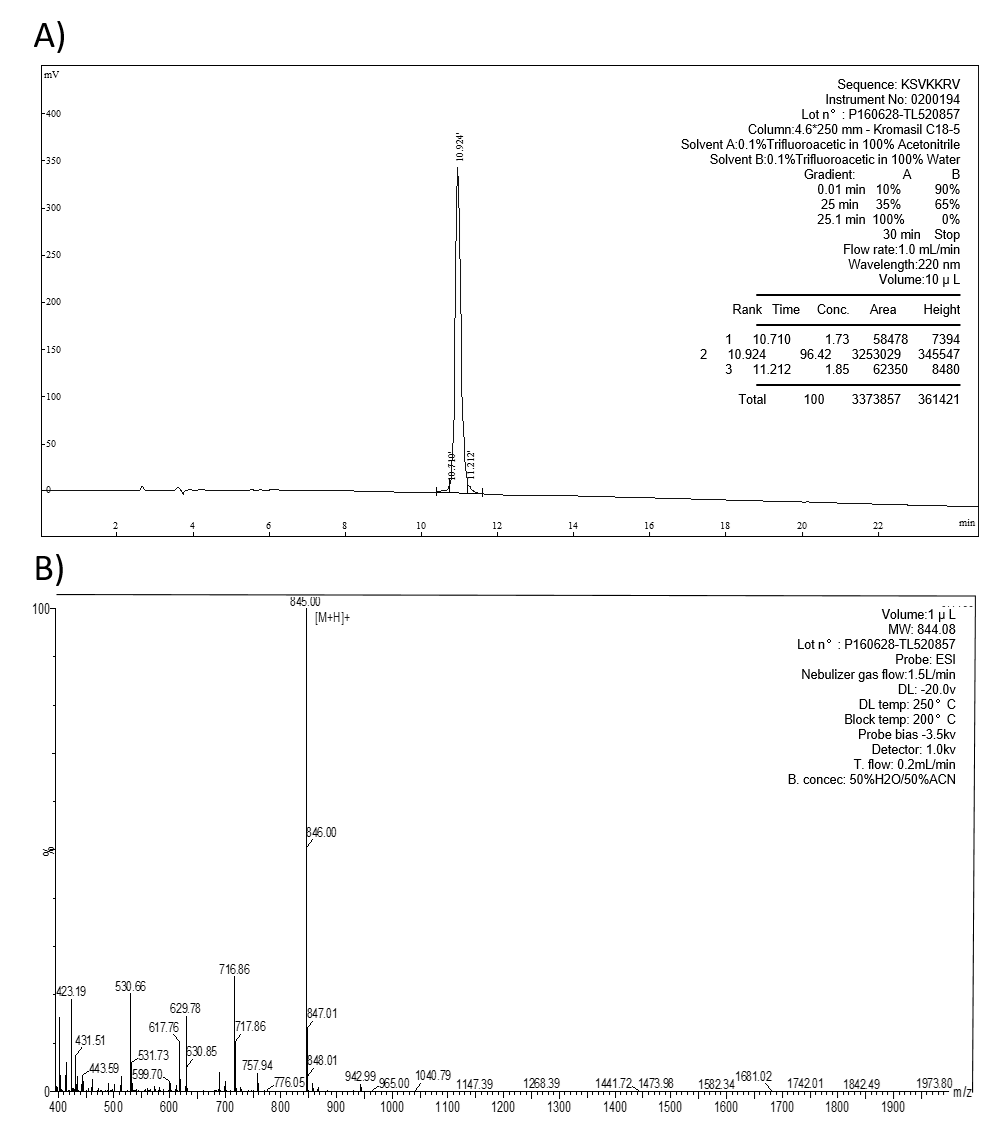

Supplement: Supplementary file 15 — Additional file 15. Peptide 6: A) HPLC analysis chromatogram. B) Mass spectrum. [file 12866_2020_1921_MOESM15_ESM.tif]

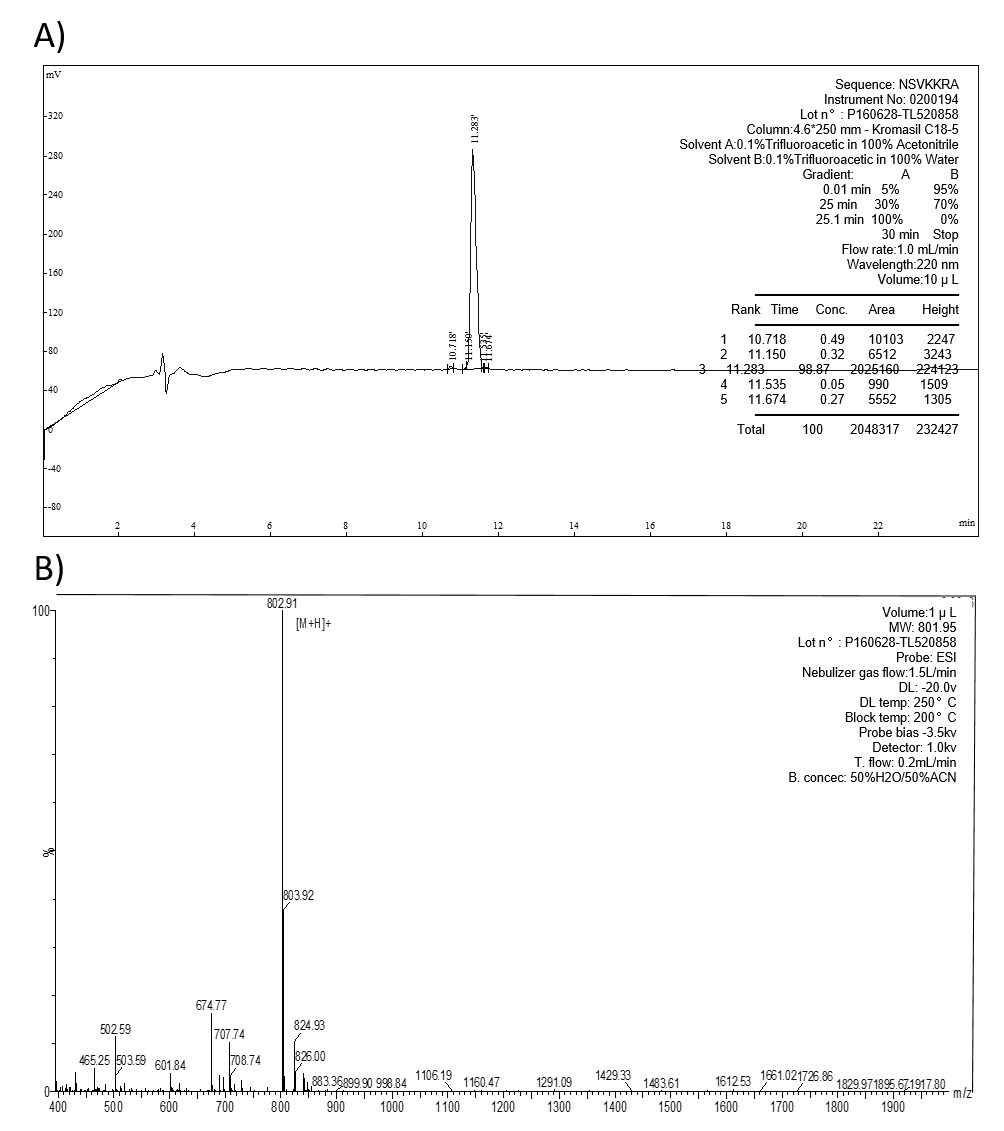

Supplement: Supplementary file 16 — Additional file 16. Peptide 7: A) HPLC analysis chromatogram. B) Mass spectrum. [file 12866_2020_1921_MOESM16_ESM.tif]

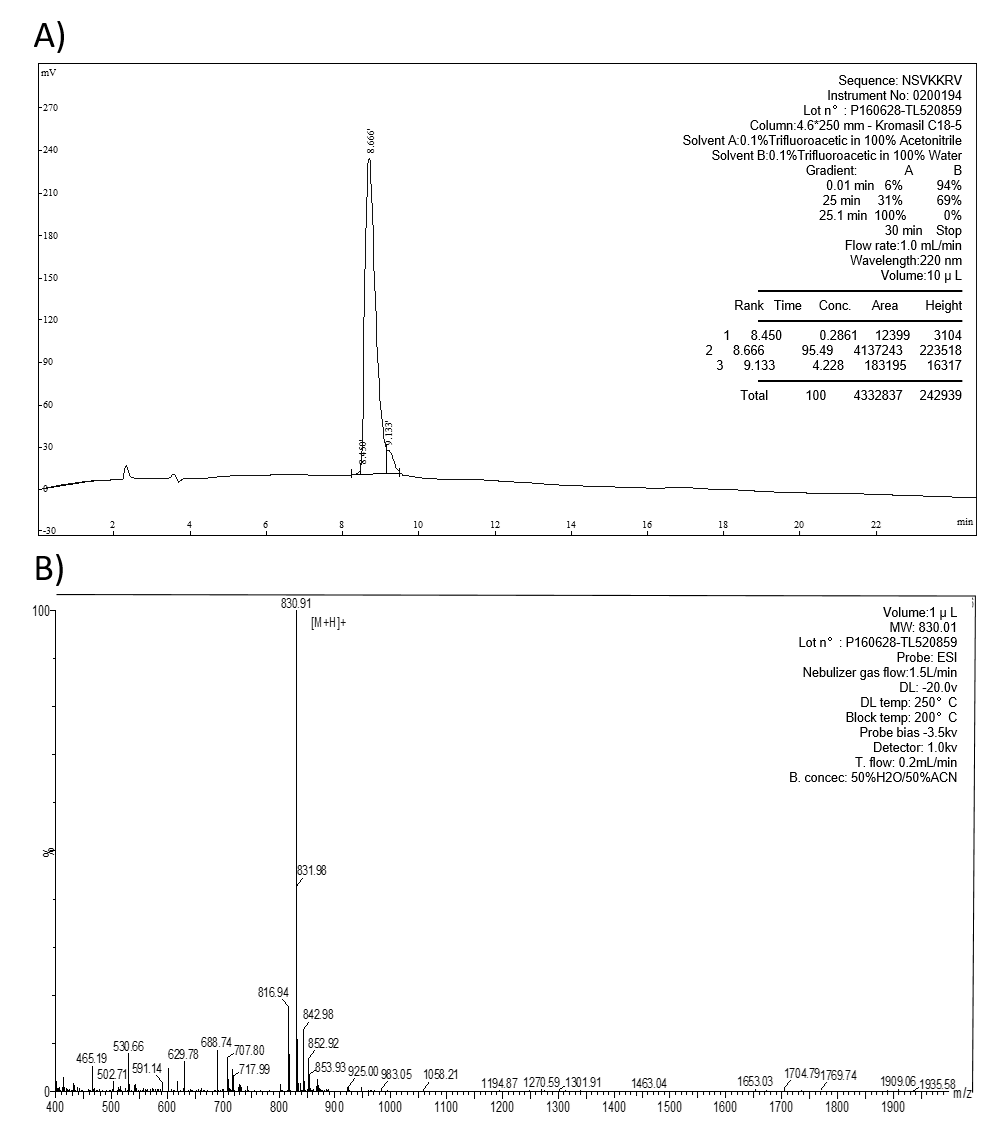

Supplement: Supplementary file 17 — Additional file 17. Peptide 8: A) HPLC analysis chromatogram. B) Mass spectrum. [file 12866_2020_1921_MOESM17_ESM.tif]

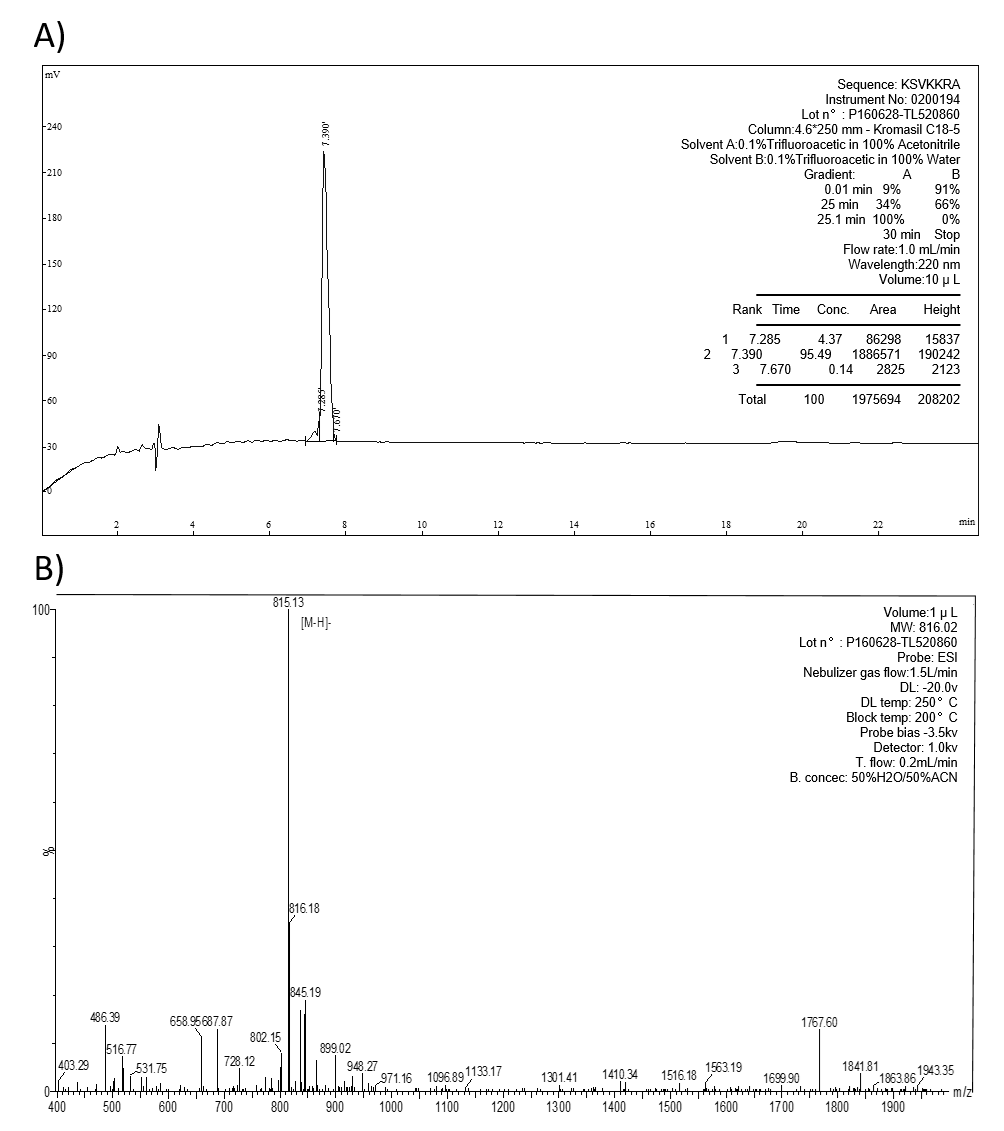

Supplement: Supplementary file 18 — Additional file 18. Peptide 9: A) HPLC analysis chromatogram. B) Mass spectrum. [file 12866_2020_1921_MOESM18_ESM.tif]

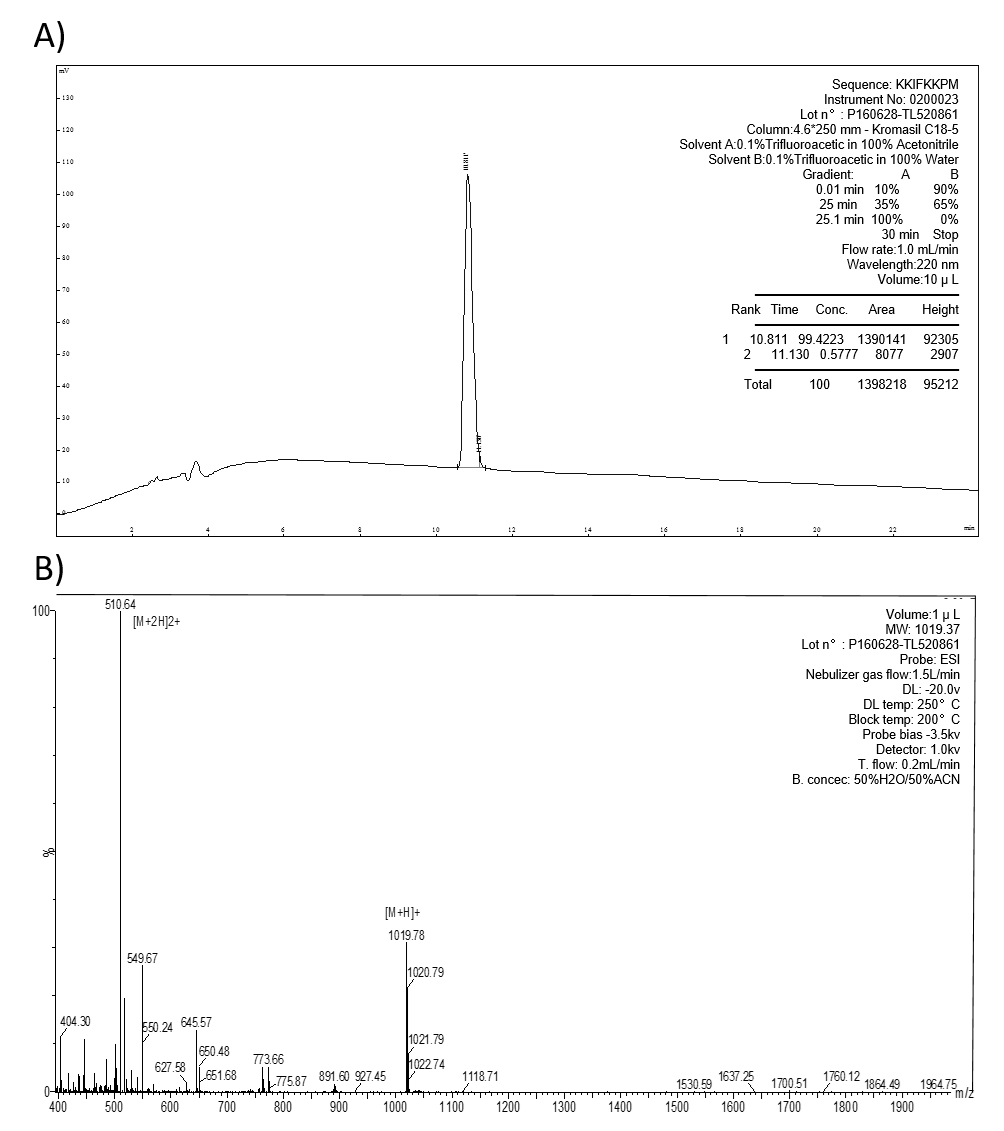

Supplement: Supplementary file 19 — Additional file 19. Peptide 10: A) HPLC analysis chromatogram. B) Mass spectrum. [file 12866_2020_1921_MOESM19_ESM.tif]

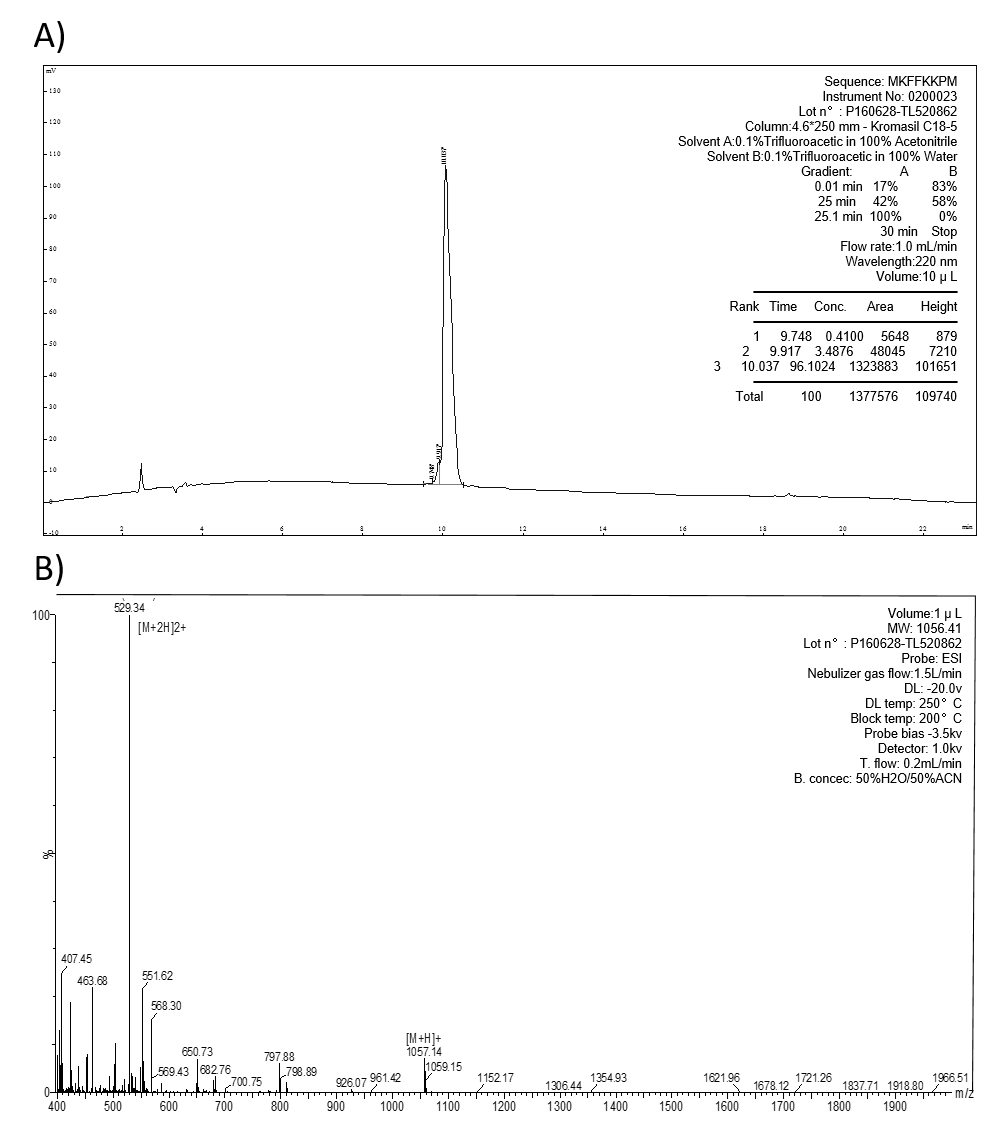

Supplement: Supplementary file 20 — Additional file 20. Peptide 11: A) HPLC analysis chromatogram. B) Mass spectrum. [file 12866_2020_1921_MOESM20_ESM.tif]

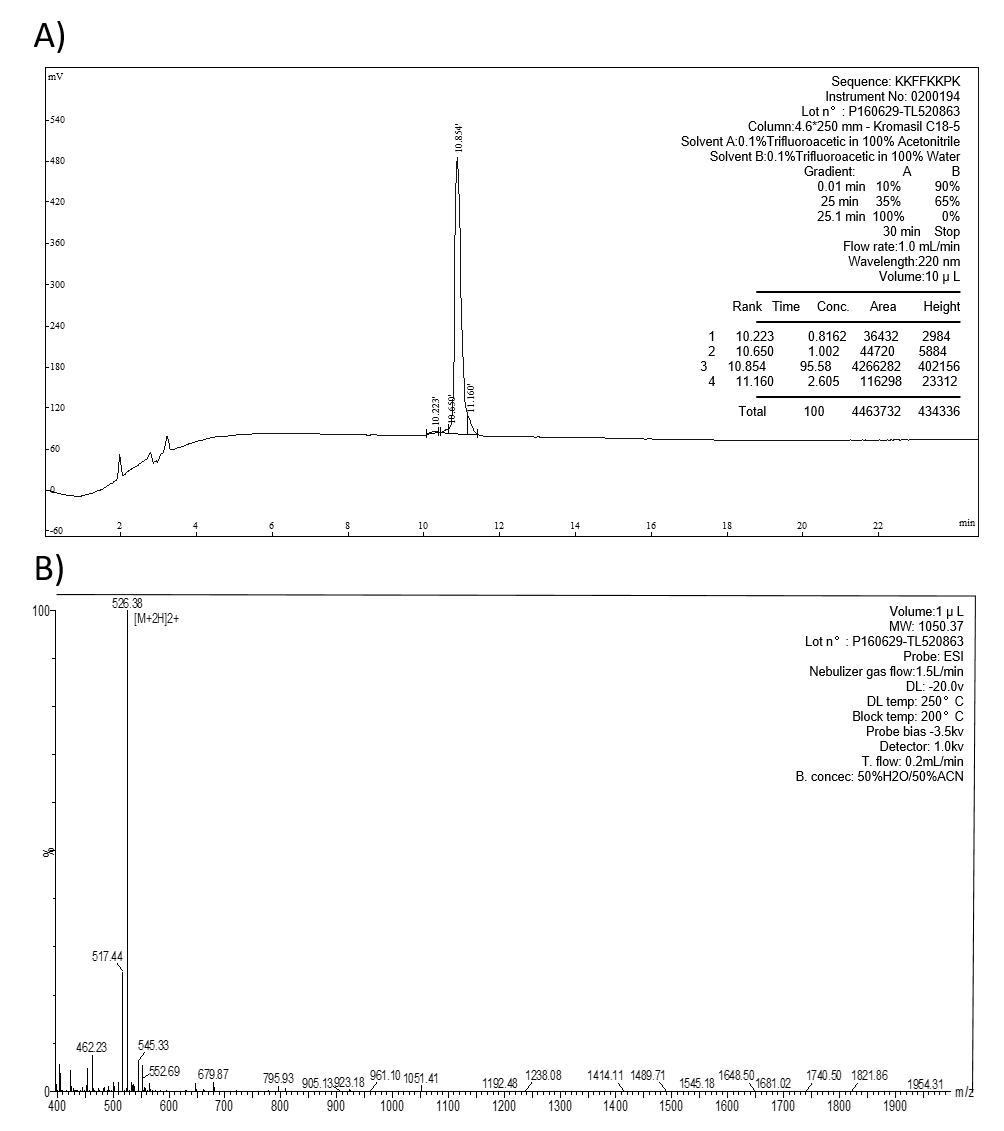

Supplement: Supplementary file 21 — Additional file 21. Peptide 12: A) HPLC analysis chromatogram. B) Mass spectrum. [file 12866_2020_1921_MOESM21_ESM.tif]

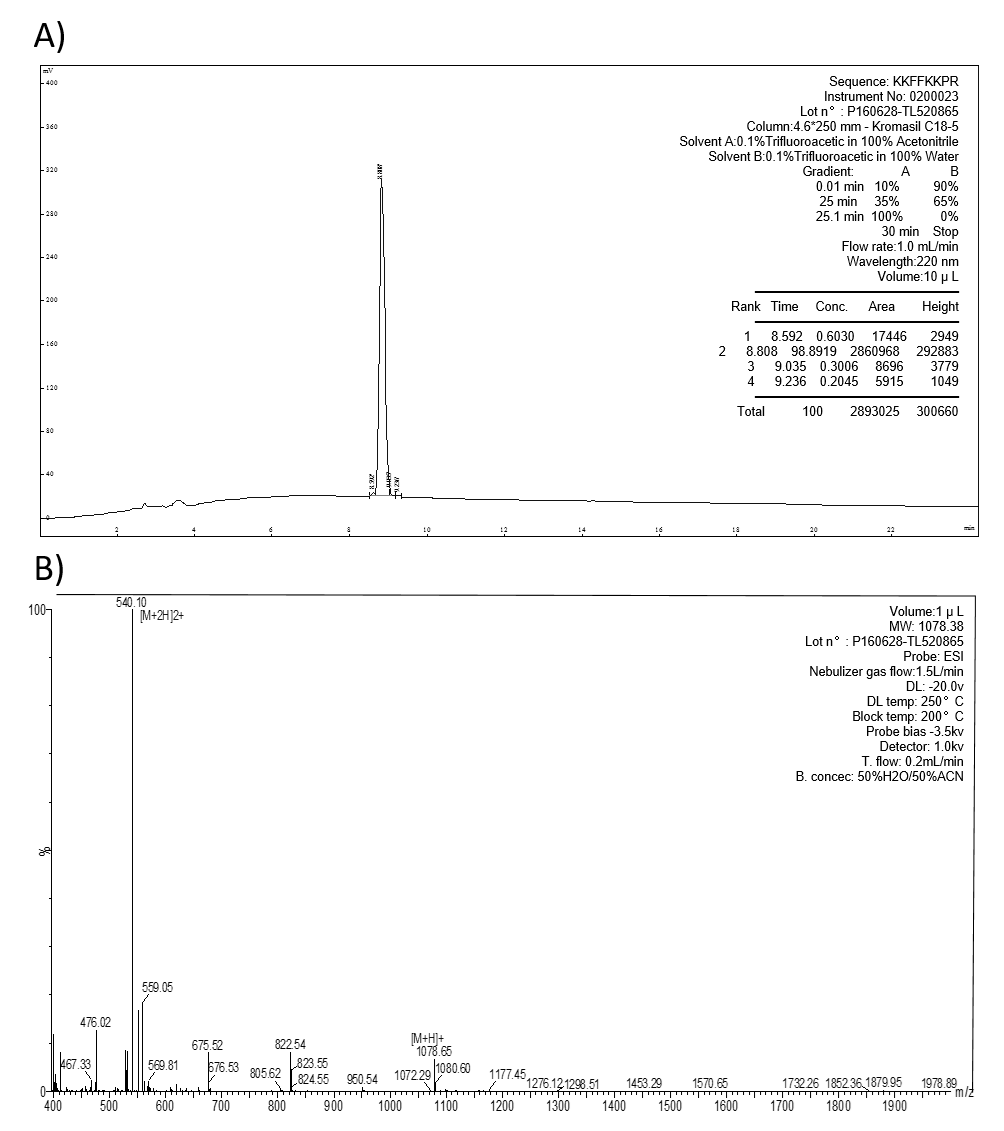

Supplement: Supplementary file 22 — Additional file 22. Peptide 13: A) HPLC analysis chromatogram. B) Mass spectrum. [file 12866_2020_1921_MOESM22_ESM.tif]

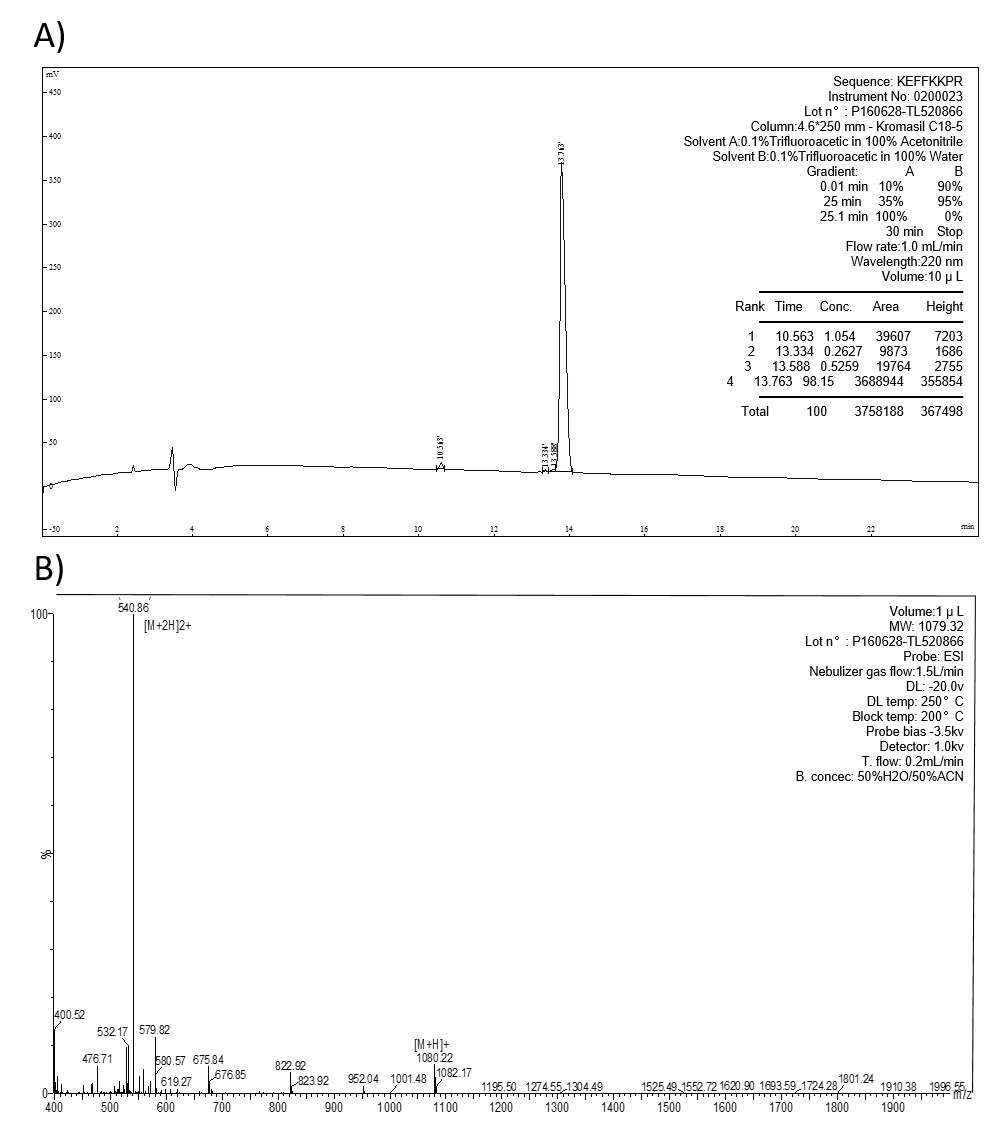

Supplement: Supplementary file 23 — Additional file 23. Peptide 14: A) HPLC analysis chromatogram. B) Mass spectrum. [file 12866_2020_1921_MOESM23_ESM.tif]

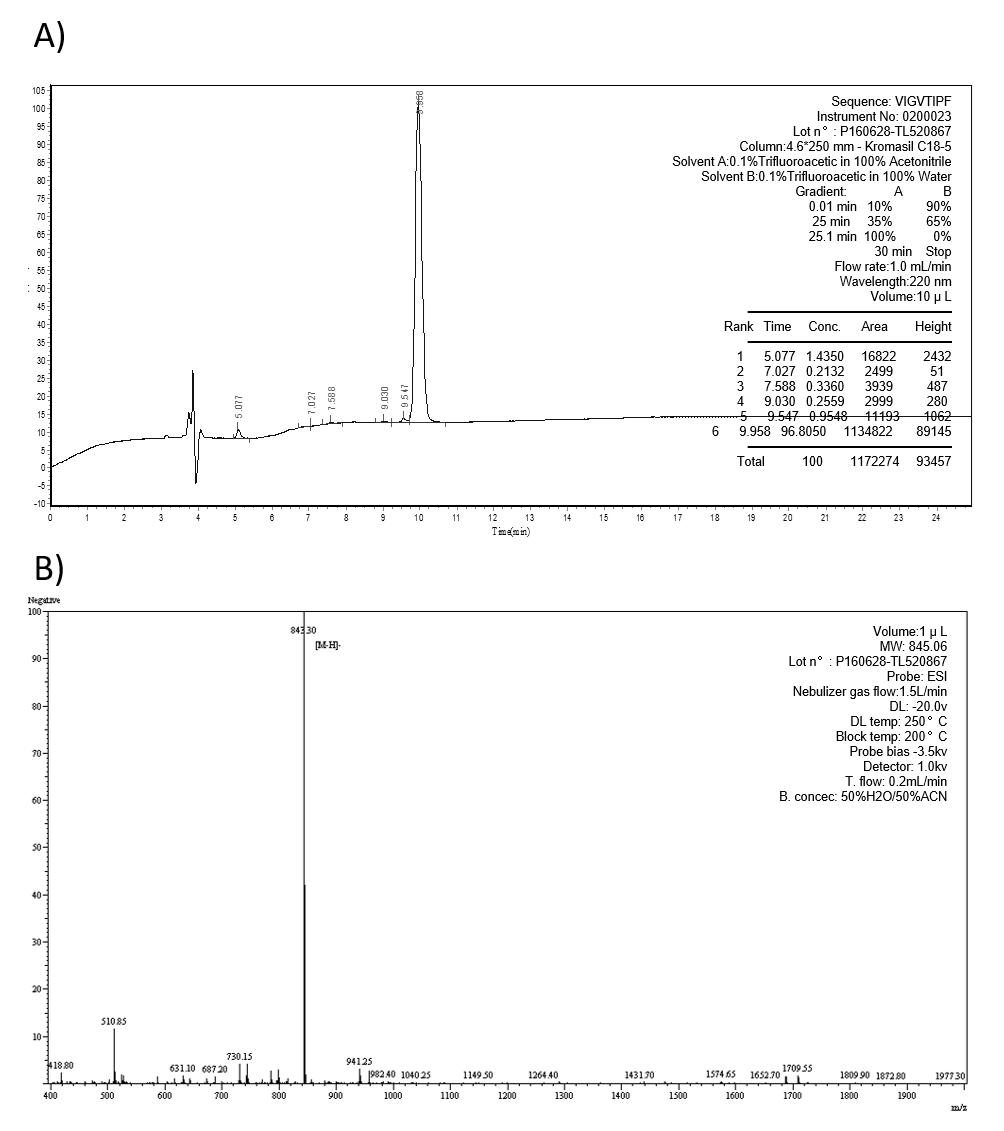

Supplement: Supplementary file 24 — Additional file 24. Peptide 15: A) HPLC analysis chromatogram. B) Mass spectrum. [file 12866_2020_1921_MOESM24_ESM.tif]

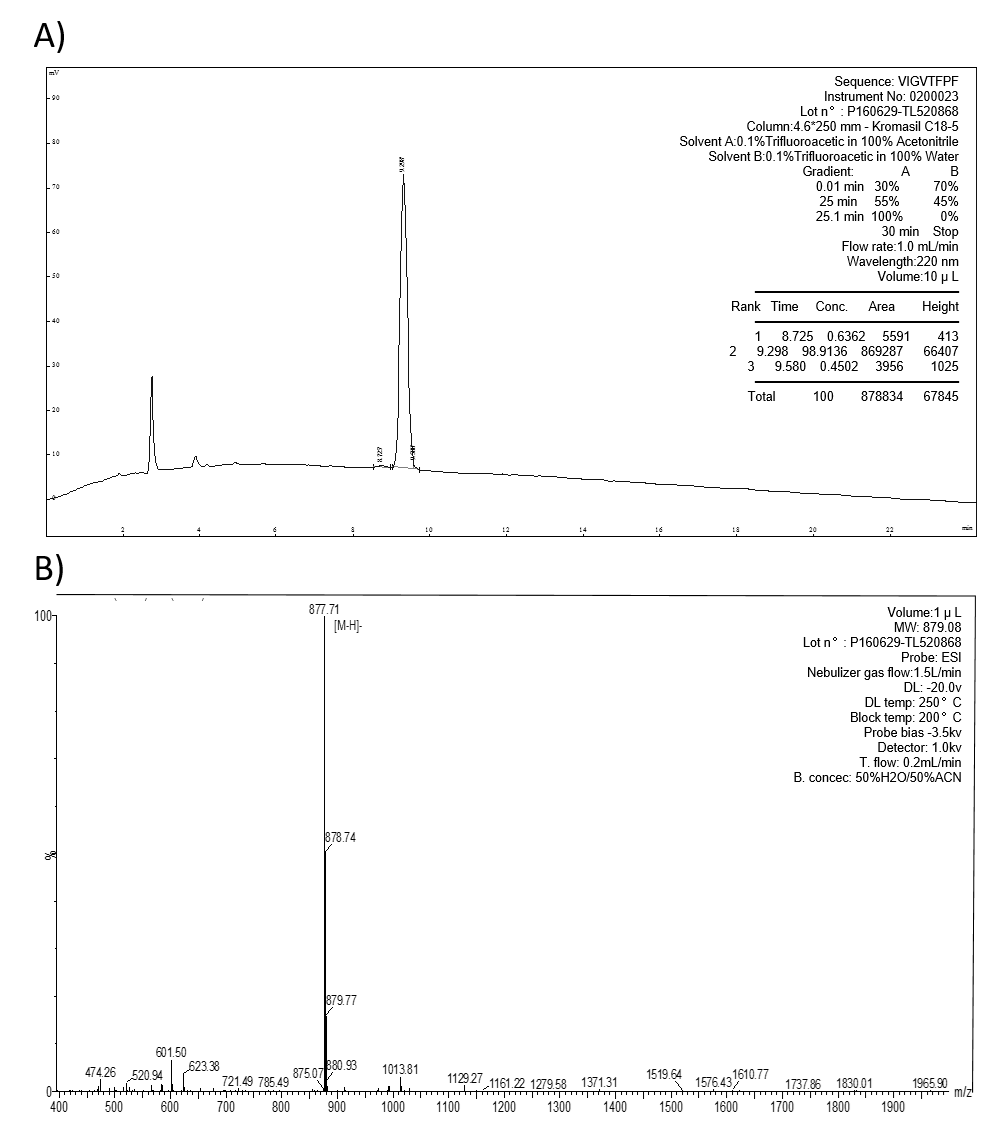

Supplement: Supplementary file 25 — Additional file 25. Peptide 16: A) HPLC analysis chromatogram. B) Mass spectrum. [file 12866_2020_1921_MOESM25_ESM.tif]

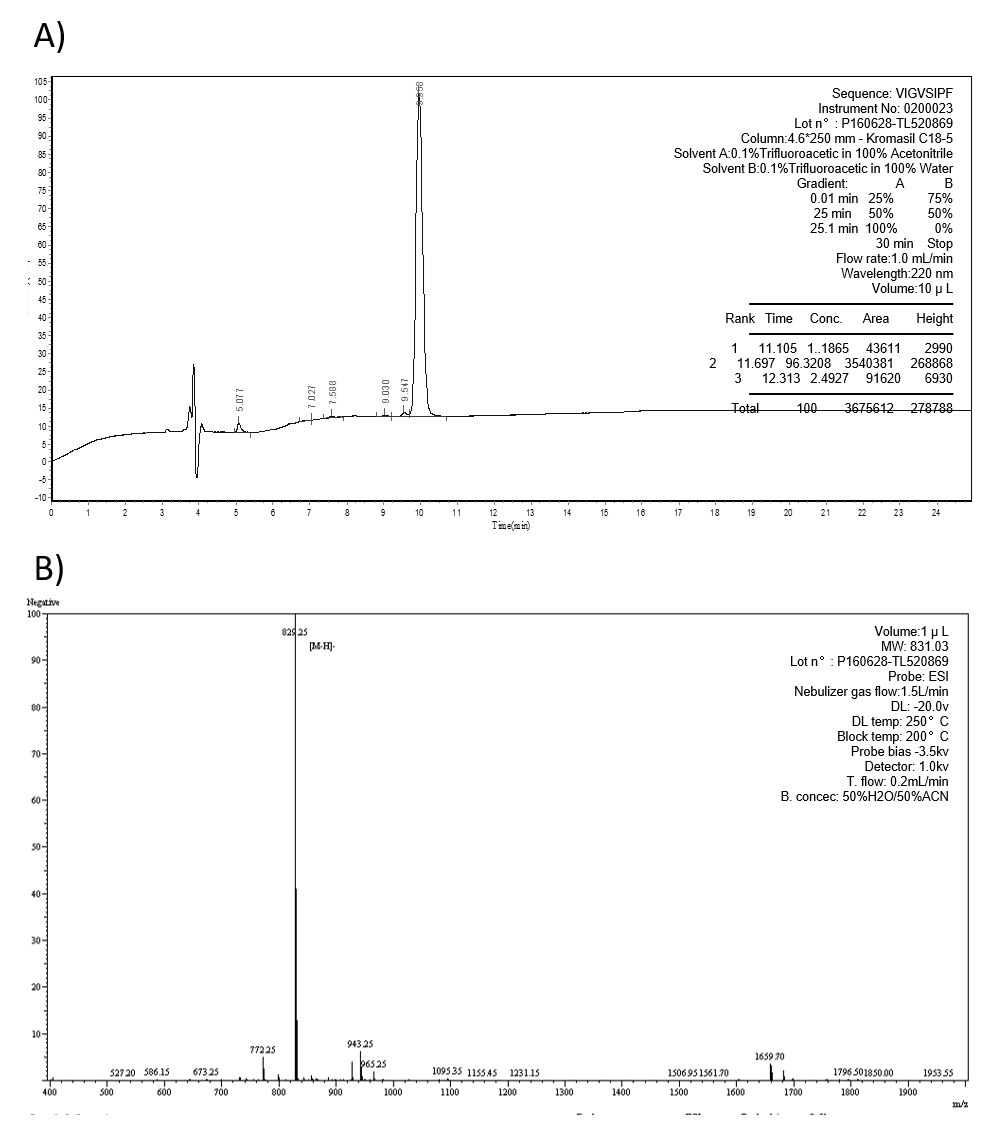

Supplement: Supplementary file 26 — Additional file 26. Peptide 17: A) HPLC analysis chromatogram. B) Mass spectrum. [file 12866_2020_1921_MOESM26_ESM.tif]
